# Repeated and Time-Correlated Morphological Convergence in Cave-Dwelling Harvestmen (Opiliones, Laniatores) from Montane Western North America

**DOI:** 10.1371/journal.pone.0010388

**Published:** 2010-05-07

**Authors:** Shahan Derkarabetian, David B. Steinmann, Marshal Hedin

**Affiliations:** 1 Department of Biology, San Diego State University, San Diego, California, United States of America; 2 Department of Zoology, Denver Museum of Nature and Science, Denver, Colorado, United States of America; Smithsonian Institution National Zoological Park, United States of America

## Abstract

**Background:**

Many cave-dwelling animal species display similar morphologies (troglomorphism) that have evolved convergent within and among lineages under the similar selective pressures imposed by cave habitats. Here we study such ecomorphological evolution in cave-dwelling Sclerobuninae harvestmen (Opiliones) from the western United States, providing general insights into morphological homoplasy, rates of morphological change, and the temporal context of cave evolution.

**Methodology/Principal Findings:**

We gathered DNA sequence data from three independent gene regions, and combined these data with Bayesian hypothesis testing, morphometrics analysis, study of penis morphology, and relaxed molecular clock analyses. Using multivariate morphometric analysis, we find that phylogenetically unrelated taxa have convergently evolved troglomorphism; alternative phylogenetic hypotheses involving less morphological convergence are not supported by Bayesian hypothesis testing. In one instance, this morphology is found in specimens from a high-elevation stony debris habitat, suggesting that troglomorphism can evolve in non-cave habitats. We discovered a strong positive relationship between troglomorphy index and relative divergence time, making it possible to predict taxon age from morphology. Most of our time estimates for the origin of highly-troglomorphic cave forms predate the Pleistocene.

**Conclusions/Significance:**

While several regions in the eastern and central United States are well-known hotspots for cave evolution, few modern phylogenetic studies have addressed the evolution of cave-obligate species in the western United States. Our integrative studies reveal the recurrent evolution of troglomorphism in a perhaps unexpected geographic region, at surprisingly deep time depths, and in sometimes surprising habitats. Because some newly discovered troglomorphic populations represent undescribed species, our findings stress the need for further biological exploration, integrative systematic research, and conservation efforts in western US cave habitats.

## Introduction

Cave habitats have long interested evolutionary biologists and ecologists – such habitats combine *geographic isolation*, promoting speciation and endemicity, with *selective similarities*, promoting convergence in life history and morphological form [1]. This combination of divergence and selective constraints leads to evolutionary trends that are predictable and often replicated independently within and among animal lineages, making cave organisms excellent candidates for comparative evolutionary analyses [2]–[6]. An example is the study of ecomorphological evolution, where ecomorphs are defined as ecological forms characterized by suites of phenotypic traits that have some conspicuous correlation with ecological attributes [7]. Cave ecomorphs are called *troglomorphs* [8], a term coined to describe a common morphological syndrome displayed by cave animals (e.g., pale, reduced eyes, elongate appendages, etc). As summarized by Culver et al., [1], “*troglomorphs are forms where the body is clearly modified for cave existence …. which are quite different from all normal cavernicolous animals. Such forms are, on the whole, easily identifiable as cave animals and have many common traits even when they belong to different taxonomic groups*”.

In continental North America, areas of high diversity for terrestrial troglomorphs include the Appalachian and Interior Lowlands regions, the central Texas uplands, and the California “Mother Lode” region [9, fig. 2]; [10]. These regions are home to terrestrial cave-obligate faunas that are species-rich and highly-endemic, reflecting a combination of habitat availability (i.e., many caves), habitat persistence combined with periodic isolation, and availability of source faunas for cave colonization and subsequent evolution. In comparison, much less has been written about the Great Basin and Rockies as a hotspot for cave evolution. Examples of exceptions include papers by Peck [11], [12], and Shear and colleagues [13]. In general, western states (except for the CA Mother Lode region) may have fewer documented cave-obligate species because there are fewer regional biospeleologists, fewer source lineages, or cave habitats less favorable for troglomorphic evolution (e.g., caves that are smaller, younger, drier, shallower, nutrient-poor, etc). Surface conditions over evolutionary time may also play a role, with periodic gene flow from surface forms swamping incipient evolution of troglomorphs.

**Figure 1:**
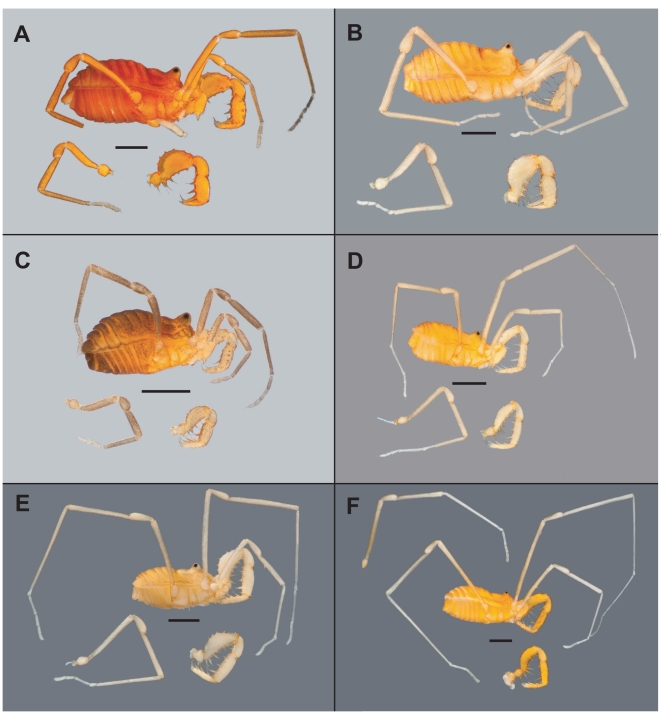
Representative sclerobunine morphological diversity. A) *S. r. robustus* (Apex Valley Road); B) *S. r. robustus* (Fault Cave); C) *S. r. glorietus* (Taos); D) *S. r. glorietus* “trog” (Taos); E) *Sclerobunus sp* (Cave of the Winds); F) *C. u. ungulatus* (Model Cave). All scale bars  = 1 mm.

Here we describe the discovery of a fascinating pattern of recurrent troglomorphism in harvestmen from the southern Rockies and adjacent western states of North America. Laniatorean harvestmen, members of the arachnid order Opiliones, are conspicuous predators in caves throughout the world [14]–[17] including North America. Over 50 species representing more than a dozen Laniatorean genera are found in North American caves, and many of these taxa are obviously troglomorphic, with pale integuments, long legs and palps, and reduced or absent eyes [6], [18]–[25]. These troglomorphic taxa also often have very small geographic distributions, and three species are listed as federally-endangered [26], [27]. This study concerns Sclerobunine harvestmen, cryophilic arachnids from western North America that are phylogenetically placed in a larger Holarctic clade, the Travunioidea [28], [29]. Three genera are currently classified as Sclerobunines, including *Cyptobunus*, *Zuma*, and *Sclerobunus*. Members of all three genera prefer moist, dark, surface microhabitats (e.g., under rocks and logs), but are sometimes found in caves. For example, all described *Cyptobunus* taxa are known only from caves, and are distinguished from other sclerobunines in elaboration of troglomorphic features (Table 1, Fig. 1). Cavernicolous *Sclerobunus* are also known from museum collections, but have never been formally studied – whether or not these troglomorphic *Sclerobunus* are independent from or phylogenetically-related to *Cyptobunus* is unknown.

**Table 1:** Taxonomic Summary of the Sclerobuninae.

Genus	Species	Geographic distribution	Distinguishing Features
*Zuma*Goodnight and Goodnight, 1942	*Z. acuta*Goodnight and Goodnight, 1942	coastal central CA	Integument yellow-brown, with some black pigment
	*Z. tioga*Briggs, 1971a	Yosemite NP, CA	Integument yellow, without black pigment
*Cyptobunus*Banks, 1905	*C. cavicolens*Banks, 1905	Lewis and Clark Caverns, MT	Troglomorphic, some black pigment, with reduced lateral branches
	*C. u. ungulatus*Briggs, 1971a	caves in Great Basin NP, NV	Highly troglomorphic, absent branches
	*C. u. madhousensis*Briggs, 1971a	Madhouse and Professor Buss Caves, UT	Highly troglomorphic, absent branches, large cornea, retina displaced
*Sclerobunus*Banks, 1898	*S. nondimorphicus*Briggs, 1971a	WA, OR, and BC	Palpal femur of males not swollen
	*S. r. idahoensis*Briggs, 1971a	ID, southern BC	Scute with much black pigment
	*S. r. glorietus*Briggs, 1971a	northern NM	Scute with black pigment,<2 mm
	*S. r. robustus*Packard, 1877	AZ, CO, NM, UT	Scute with some black pigment, >2 mm

**Notes:** BC, British Columbia; CA, California; ID, Idaho; MT, Montana; NV, Nevada; OR, Oregon; UT, Utah; WA, Washington.

In this paper we examine internal relationships of the Sclerobuninae using DNA sequence data from three independent gene regions. These gene tree data are combined with Bayesian hypothesis testing, morphometrics analysis, study of penis morphology, and relaxed molecular clock analyses to understand the biogeography and timing of troglomorphic evolution within this group. Central questions include the prevalence of homoplasy in troglomorphism, the age of troglomorphic taxa, and the geographic context of this evolution. We find that troglomorphic populations/species have evolved multiple (3–5) times independently within sclerobunines, are relatively old, and generally have small geographic distributions. Some of these troglomorphic populations almost certainly represent undescribed species deserving conservation attention. One troglomorphic population is found in a high-elevation non-cave situation, suggesting that troglomorphs sometimes evolve in habitats other than caves. Overall, this study reveals a surprisingly dynamic picture of cave evolution in montane western North America.

## Materials and Methods

### Taxon Sampling

Most of the specimens used in this study were collected in recent fieldwork conducted by the authors - when collecting a series of individuals, some were preserved in 80% EtOH for morphological analysis, whereas specimens destined for molecular analysis were preserved in 100% EtOH (and later stored at −80 C). Other specimens used in both molecular and morphological analysis were borrowed from museums (see Acknowledgements).

The clade Travunioidea has recently been recovered in phylogenetic analyses of Laniatores. The monophyly of this group is supported by both morphological features [28] and molecular data [29]. Travunioids comprise three Holarctic families (Travuniidae, Cladonychiidae, and Briggsidae), and several northern hemisphere subfamilies that were recently transferred from the Triaenonychoidea (Sclerobuninae, Paranonychinae, Kaolinonychinae, and Nipponychinae). Within this larger group, the northern hemisphere triaenonychids may form a clade, based on the musculature of the penis, and the trident shaped tarsal claw with variable numbers of side branches [30]. As outgroups for this study we included North American taxa representing the Travuniidae (*Speleonychia*), Cladonychiidae (*Erebomaster, Cryptomaster, Theromaster*, *Speleomaster*), Briggsidae (*Briggsus*), and the Paranonychinae (*Metanonychus* and *Paranonychus*). *Fumontana deprehendor* and *Equitius doriae* were used as distant outgroups to Travunioidea, following results of Giribet et al., [29].

All described sclerobunine taxa were represented in our sample (Table S1). This includes two narrowly-distributed *Zuma* species from California, and all described taxa (species and subspecies) of both *Cyptobunus* and *Sclerobunus*. The species *Sclerobunus robustus* includes two relatively widespread subspecies (*S. r. robustus* and *S. r. glorietus*), which may contain cryptic lineages – we first conducted a comprehensive mitochondrial phylogeographic survey of the entire geographic distribution of each subspecies [31], and include here geographic representatives from all primary mitochondrial lineages.

**Figure 2:**
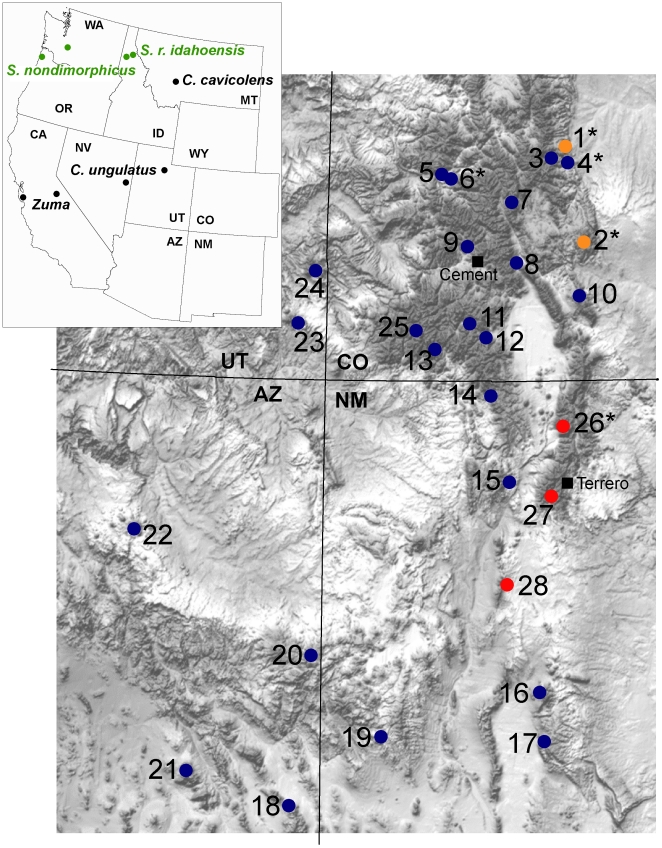
Distribution of the Sclerobuninae, emphasizing sampling locations of *Sclerobunus robustus*. Colors correspond to clades recovered in combined analysis. Map numbers as in Table S1.

We emphasized the sampling of cave populations. Two species of *Cyptobunus* were sampled from geographically-isolated caves in UT, NV and MT. As discussed above, *Cyptobunus* taxa are known only from caves (Table 1), are obviously cave-modified (Fig. 1), and lack *known* neighboring surface populations (Fig. 2). We also included four newly-discovered cave-dwelling populations of *S. robustus* from Colorado caves (Cave of the Winds, Mallory Cave, Fault Cave, Skeleton Cave). *A priori*, we qualitatively classified these populations as troglomorphic based on overall morphological appearance. These cave populations are less geographically isolated from nearby surface populations, and in some instances are adjacent to forests with surface-dwelling *Sclerobunus*. Finally, we sampled from a high-elevation montane site in north-central New Mexico (near Taos) where we discovered qualitatively troglomorphic animals from deep stony debris (0.25–1 meter below the surface), syntopic with “typical” specimens collected closer to the surface (see Fig. 1). We are not aware of cave habitats near this montane location.

### Morphometrics Analysis

Linear measurements of the body, eye mound, chelicerae, palps, and the first two legs were taken using a dissecting microscope fitted with an ocular micrometer (Table S2). Characters chosen for analysis have been used in other studies of laniatorean morphology [32]. All measurements were taken from adult males, except for the inclusion of female specimens from Taos (both surface and troglomorphic forms), Skeleton and Fault Caves, and their respective nearest surface locations (Hanging Lake and Apex Valley Road). We also measured specimens of slightly troglomorphic *Sclerobunus* from Terrero Cave (NM) and Cement Creek Cave (CO), but were unable to acquire molecular data for these cave populations.

We conducted a Principal Components Analysis (using Systat 11, Systat Software, Inc.) to assess whether cave-derived samples (plus deep stony debris Taos specimens) consistently occupy a distinct region of multivariate morphological space. Based on results of PCA analyses, we *quantitatively* classified populations/taxa as troglomorphic. We then used Bayes factor analyses to test several alternative hypotheses regarding the evolution of troglomorphism in the context of molecular phylogenetic data (see Table 2). We used the “prset topologypr” setting in MrBayes, and set the prior probability of the constrained group to 100 [33]. The Bayes factor (BF) was calculated by taking twice the difference of the –lnL harmonic means between unconstrained and constrained analyses [34], and interpreted following Kass and Raftery [35].

**Table 2:** Results of Bayes Factor hypothesis testing.

Constraint	-lnL Harmonic mean (post burnin)	Bayes Factor	Support for Rejection
No constraint	−25191.16	--	--
Sclerobuninae monophyletic	−25202.24	22.16	Very Strong
All troglomorphs monophyletic (single origin)	−25446.55	510.78	Very Strong
*Cyptobunus* separate from troglomorphic *Sclerobunus* (two origins)	−25286.94	191.56	Very Strong
*Cyptobunus*, COW+Mallory, Taos, Fault+Skeleton Cave (four origins)	−25214.46	46.6	Very Strong

**Notes:** Single origin hypothesis - all cave-modified samples (including *Cyptobunus* and troglomorphic *Sclerobunus*) constrained to form a clade. Two origins hypothesis - troglomorphic *Sclerobunus* constrained to form a clade independent of *Cyptobunus*. Four origins hypothesis - troglomorphic *Sclerobunus* from Fault and Skeleton Caves constrained to form a clade, independent of other troglomorphic *Sclerobunus* and *Cyptobunus*.

### Molecular Data Collection

Genomic DNA was extracted from leg tissue using the Qiagen DNeasy Kit, per manufacturer's protocol. Three separate gene regions were PCR-amplified and sequenced – these included portions of the mitochondrial cytochrome oxidase I (CO I) gene, the nuclear ribosomal 28S gene, and the nuclear elongation factor I alpha (EF-1α) gene (see Table S3 for primers). EF-1α sequences were generated for a reduced taxon sample that did not include all travunioid outgroups. Amplifications followed previously-published methods [6], [36], [37], except that a MasterAmp kit (Epicentre Biotechnologies) was used for some 28S reactions, and some EF-1α reactions were conducted in a nested fashion, using amplification products from an initial reaction in a second reaction with higher annealing temperatures. PCR products were purified using Polyethylene Glycol (PEG) precipitation or gel purification (QIAquick Gel Extraction Kit, QIAGEN), and sequenced using Big Dye Version 3 dye chemistry (ABI). All DNA sequencing was performed at the San Diego State University Microchemical Core Facility.

### Phylogenetic Analysis

Contiguous sequences were assembled from bi-directional reads and manually aligned using Sequencher 4.5 (Gene Codes Corporation, MI) and MacClade v 4.06 [38]. The CO1 alignment included 3-bp indels in *Fumontana* and *Equitius*; these were manually aligned, guided by amino acid translations. The length variable 28S data was first aligned using default parameters in ClustalX v 1.83 [39], then manually adjusted. The EF-1α sequences included both exon data (aligned manually), and a rapidly evolving intron; this intron was difficult to align across the divergent taxon sample, and was therefore removed from phylogenetic analyses.

Both unpartitioned and partitioned Bayesian analyses of combined data were used to infer phylogenetic relationships, conducted using the software MrBayes 3.0b4 [40]. Models used in analyses were chosen with MrModeltest [41]. Several different partitioning strategies were tested using BF analyses to determine an optimal partitioning strategy (Table S4). As a convergence diagnostic, all analyses were permitted to run until the standard deviation of split frequencies fell below 0.01 [33]. After discarding the first 40% of tree topologies as burn-in, topologies were summarized as a majority-rule consensus tree. Taxon bipartitions with posterior probabilities over 95% are considered strongly supported. Bayes factor analysis (as described above) was used to test the monophyly of the Sclerobuninae (i.e., *Zuma*, *Sclerobunus*, and *Cyptobunus* were constrained to form a monophyletic group).

### Divergence Time Estimates

The concatenated matrix was analyzed using BEAST software [42], [43], which uses a Metropolis-Hastings MCMC algorithm to simultaneously estimate evolutionary parameters, phylogeny, and divergence dates under a relaxed clock model. Relaxed clock methods using BEAST have been used to estimate the timing of troglomorphic evolution in other cave-dwelling taxa [5], [44]–[46]. Data were partitioned by gene (CO1, 28S, EF-1α) and by codon position (CO1 and EF-1α) using the same models selected for each partition as in MrBayes analyses, and run twice (to assess convergence) for 30 million generations with 50% burnin. Use of an uncorrelated relaxed clock branch rate model allows for variable rates of molecular evolution among branches. Both fossil and biogeographic evidence was used to place clock calibrations (see Results).

### Penis Morphology

Male genitalia are often the most conspicuously different characters observed when comparing closely-related terrestrial arthropod taxa [47]. This is also often the case for laniatoreans, where studies of male penis morphology are stressed in the taxonomy of closely-related taxa [48]. For a representative sample of sclerobunines and close outgroups, male penises were extracted by pushing the penis through the genital operculum with an insect pin inserted through the opening of the anal operculum. The penis was then soaked in a cold solution of 10% KOH, and slide-mounted using a small amount of KY-Jelly immersed in filtered 70% EtOH. Photographs were taken with a Visionary Digital imaging system (http://www.sci.sdsu.edu/eb/imaging.html). Individual images were compiled into a composite image using Helicon Focus (http://www.heliconsoft.com/heliconfocus.html) and edited using Adobe Photoshop CS3.

## Results

### Data Availability

Voucher specimens for most samples used in DNA analyses are currently housed in the SDSU Terrestrial Arthropods collection – these specimens will ultimately be deposited at the California Academy of Sciences. Other DNA specimens reside at the Denver Museum of Nature and Science. All DNA sequences have been deposited to GenBank (accession numbers in Table S1). A Google Earth kmz file is available upon request from the corresponding author.

### Morphometrics Analysis

**Figure 3:**
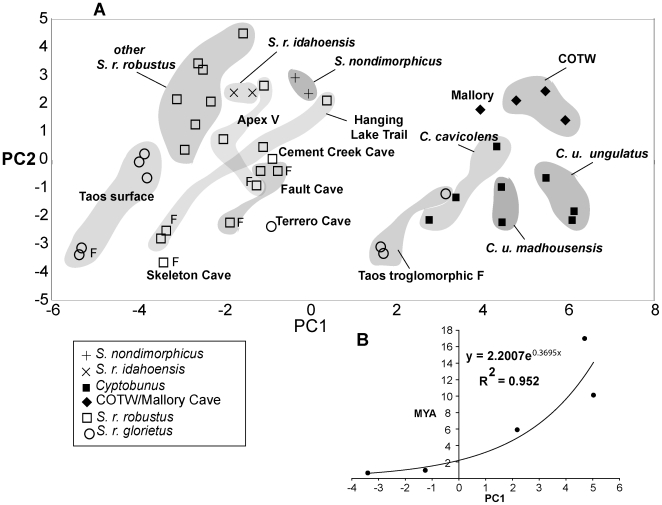
Results of principal components analysis, and morphology versus time relationship. Panel A: Principal components scatterplot of size-corrected data. Unless otherwise indicated (F), all specimens are male. Panel B: Relationship between “troglomorphy index” and divergence time estimates for troglomorphic populations/taxa.

To control for body size, a PCA was run in which the raw data was scaled by scute length, which is an appropriate measure of overall size. This analysis resulted in two principal components accounting for 76% of the total variation (PC1 –53.8, PC2 –22.2). PC1 accounts for much of the overall length of the specimen, specifically leg length, and PC2 accounts for palpal and cheliceral size (based on component loadings, not shown). The size-scaled PCA reveals sexual dimorphism, where male specimens load more positively on PC2 (e.g., Taos surface male versus female, Hanging Lake Trail male versus female, see Fig. 3A). Cave-derived samples of *Cyptobunus*, and *Sclerobunus* from Mallory Cave and Cave of the Winds, cluster together in morphological space (Fig. 3A). Deep stony debris specimens from Taos cluster with these samples, rather than with “surface” samples from Taos. Less- modified specimens from Terrero, Cement Creek, Fault, and Skeleton caves are “intermediate” between cave versus surface morphologies. For Fault and Skeleton caves, these differ slightly from geographically-proximate surface samples that are phylogenetically-related.

### Combined Analysis Results

The combined data matrix included approximately 2900 total characters, coming from CO1 (1140 bp, some shorter), 28S (1200 bp, including gaps), and the EF-1α exon (570 bp). All three genes contributed parsimony informative (PI) characters (*Sclerobunus + Cyptobunus* only) as follows: CO1 (335 PI), 28S (49 PI), and EF-1α exon (54 PI). Each different concatenated sequence was derived from a single specimen, but not all specimens included data for all gene partitions (Table S1). EF-1α sequences could not be collected for three *Sclerobunus* samples (Ski Apache, Fault Cave, Skeleton Cave), and EF-1α outgroup sampling included only *Paranonychus* and *Zuma*.

**Figure 4:**
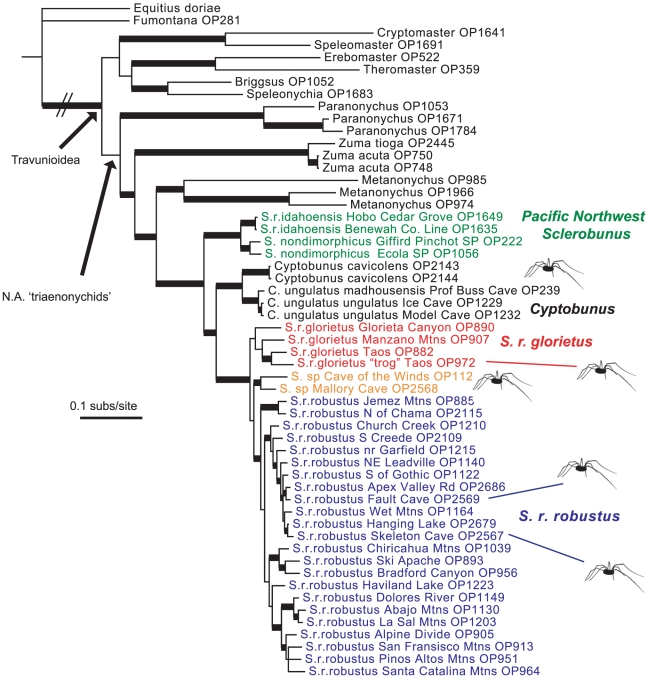
Combined-data 50% majority rule consensus Bayesian tree. Major clades are named, thick branches are supported by a posterior probability of 95 or higher. Troglomorphic populations/taxa indicated by special icons.

Analysis of combined data recovered a monophyletic Travunioidea with respect to distant outgroups (Fig. 4). The Cladonychiidae are split into eastern and western North American clades, with the eastern cladonychiids (*Erebomaster* and *Theromaster*) recovered as sister to a clade containing the western *Briggsus* and *Speleonychia*. Northern ‘triaenonychids’ are recovered as monophyletic (although with weak support), with an early-diverging *Paranonychus*, then *Zuma*, then *Metanonychus* sister to *Sclerobunus* plus *Cyptobunus*. The alternative hypothesis of a monophyletic Sclerobuninae ((*Zuma* (*Sclerobunus*, *Cyptobunus*)) is not supported by BF analysis (Table 2).

The *Sclerobunus* plus *Cyptobunus* clade is strongly-supported, with an early-diverging Pacific Northwest clade containing *S. nondimorphicus* and *S. robustus idahoensis*. Described species of *Cyptobunus* form a clade sister to *Sclerobunus robustus* (excluding *S. r. idahoensis*). The *S. robustus* clade includes three primary lineages, including the described subspecies *S. r. glorietus* and *S. r. robustus*, and a Cave of the Winds plus Mallory Cave clade. This combined analysis tree implies at least three separate origins of troglomorphy within the sclerobunines, including one transition in the ancestor of *Cyptobunus*, and two additional independent transitions in troglomorphic *Sclerobunus* from Taos (within *S. r. glorietus*), and the ancestor of Cave of the Winds plus Mallory Cave (Fig. 4). If the non-sister Fault Cave and Skeleton Cave samples (within *S. r. robustus*) are considered to be troglomorphic, then five separate origins are implied. Using BF analyses, we reject the alternative single origin, two origins, and four origins hypotheses (Table 2).

### Divergence Time Estimates

**Figure 5:**
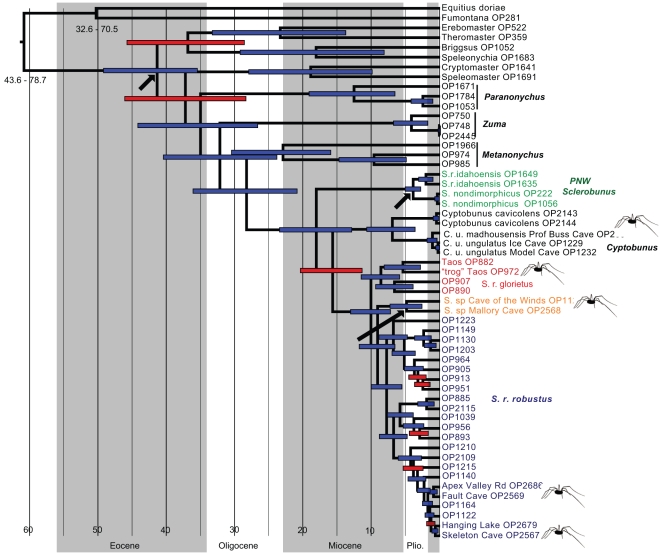
BEAST phylogram. Based on three calibration points (arrows), with 95% confidence intervals for all estimated nodes. Confidence intervals for the root of the tree and the outgroup clade are given below the nodes. Posterior probabilities less than 95 are shown with red node bars. Troglomorphic populations/taxa indicated by special icons. Time for geologic epochs from the U.S. Geological Survey Geologic Names Committee, 2007, Divisions of geologic time—Major chronostratigraphic and geochronologic units: U.S. Geological Survey Fact Sheet 2007-3015, 2 p.

Multiple calibration points were used in BEAST analyses. First, the recovered sister relationship between *S. nondimorphicus* and *S. r. idahoensis* corresponds to a well-known biogeographic break in the northern Cascades and northern Rocky Mountains [49], [50]. The estimated divergence time for disjunct taxa in these respective regions corresponds to the uplift of the Cascade Mountains, 2–5 Ma. We used a normally-distributed prior with a mean of 3.5 (95% confidence interval of 2.03–4.97 Ma). Second, a Baltic amber fossil classified as a cladonychiid [*Protoholoscotolemon*; 51], which dates from 38–54 Ma, was used as a minimum estimate for all Travunioidea (cladonychiids span the root node of Travunioidea in BEAST topologies, Fig. 5). Here we used a gamma-distributed prior, with an alpha of 2, beta of 7, and offset from zero by 35 (95% confidence interval of 38–80 Ma). A gamma distribution was used such that the node at the base of Travunioidea could be no younger than 38 Ma, but could be older, reflecting uncertainty in phylogenetic placement and date of the fossil calibration. Finally, the Cave of the Winds plus Mallory Cave split was calibrated to be no older than 5 Ma, using a lognormal distribution (mean of 1, standard deviation of 0.4). Although dating cave formation times is often difficult, it is generally agreed upon that most extant limestone caves worldwide are less than 10 million years old, with most being no more than several million years old [52], [53]. Using several dating techniques, Luiszer [54] concluded that the majority of the formation of Cave of the Winds occurred more recently than 5 Ma. We also conducted BEAST analyses without this third calibration (Fig. S1). Effective Sample Size (ESS) values for all parameters were considered to be sufficient (over 200) in all runs for both two- and three-calibration BEAST analyses.

The three-calibrations BEAST tree topology was almost identical to the combined Bayesian topology, except for minor differences within subclades (Fig. 5). A full set of date estimates is found in the Table S5 – here we stress estimated dates for troglomorphic species and populations. These time estimates are generally Pliocene or Miocene in age, including the common ancestor of *Cyptobunus* (95% HPD 3.7–10.7 Ma), and the common ancestor of syntopic forms at Taos (95% HPD 2.9–8.2 Ma). Time estimates for divergence of Fault and Skeleton cave populations from geographically-proximate surface populations are Pleistocene in age (95% HPD 0.4–1.6, 0.7–1.2, respectively). Two-calibration BEAST analyses result in similar, but slightly older time estimates (Fig. S1). In these analyses, the common ancestor of the Cave of the Winds plus Mallory clade is estimated to have diverged in the late Miocene (95% HPD 7.2–13.4 Ma).

To assess the relationship between troglomorphy and estimated divergence times, we used PC1 loadings as a proxy for troglomorphy. Because this proxy is also related to increased leg length, there exists a possible functional relationship, as the second “antenniform” leg of harvestmen is a sensory appendage [22], [55]. The correlation between PC1 loadings and estimated time since divergence from the nearest non-troglomorphic relative (mean values from two-calibration BEAST analyses) is nearly linear (correlation coefficient of 0.952, Table 3 and Fig. 3B).

**Table 3:** Values for “troglomorphy index” and corresponding divergence times.

Troglomorphic Sample	Avg PC1	Surface sister taxon	Estimated divergence time (mean)
Skeleton Cave	−3.402	Hanging Lake	0.74
Fault Cave	−1.254	Apex Valley Road	1.0
Taos	2.165	Taos	5.9
Cave of the Winds/Mallory	5.030	*S. r. robustus*	10.1
*Cyptobunus*	4.702	*S. robustus*	17

**Notes:** Time estimates from comparisons between troglomorphic samples and non-troglomorphic sister taxa. Divergence times from the two-calibrations BEAST analysis.

### Penis Morphology

**Figure 6:**
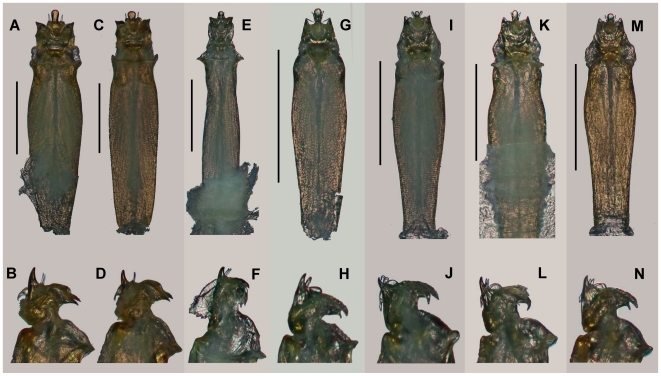
Ventral and lateral views of penis for representative samples. A, B) *S. nondimorphicus*, C, D) *S. r. idahoensis*, E, F) *C. u. ungulatus* – Model Cave, G, H) *S. r. glorietus* – Glorieta Canyon, I, J) *Sclerobunus sp.* – Cave of the Winds, K, L) *Sclerobunus sp.* – Mallory Cave, M, N) *S. r. robustus* – Hanging Lake, All scale bars  = 0.5 mm.

Twenty-two male specimens were examined, including samples for *Metanonychus*, *Paranonychus*, *Zuma*, and all *Cyptobunus* and *Sclerobunus* taxa. Consistent with molecular phylogenetic data, penis morphology suggests that *Metanonychus* is more similar to the sclerobunines than is *Paranonychus* or *Zuma* (data not shown). All *Sclerobunus* and *Cyptobunus* share a trident shaped stylus. *Sclerobunus nondimorphicus* and *S. r. idahoensis* are very similar in the fine structure, spination, and size of the penis, which is noticeably different from *S. robustus*. *Cyptobunus* taxa share a morphology that is quite different from *Sclerobunus* (Fig. 6E, F). *Sclerobunus r. glorietus* and *S. r. robustus* penises are nearly identical in structure, but differ in overall size (*S. r. robustus* being nearly 50% larger than *S. r. glorietus*). Troglomorphic males from Cave of the Winds differ from other *S. r. robustus* in the recurved spines of the stylus (Fig. 6I, J), whereas males from Mallory Cave possess a reduced stylus, somewhat resembling the stylus of *Cyptobunus* (Fig. 6K, L). There are no obvious differences in penis structure between moderately troglomorphic *S. r. robustus* (Fault Cave) and geographically-proximate surface samples (Apex Valley Road; data not shown). Males from the troglomorphic Taos population remain unknown.

## Discussion

The evolution of similar troglomorphic characteristics in distantly-related animal lineages (e.g., fish, salamanders, spiders, beetles, harvestmen, etc) clearly illustrates the power of evolutionary convergence at deep phylogenetic levels. Over the past 10 years, studies which have viewed morphological evolution through the lens provided by molecular phylogenetics have likewise revealed troglomorphic convergence *within* groups. Examples include Mexican cavefish [56], cave catfishes [57], salamanders [3], shrimp [45], amphipods [5], diving beetles [2], spiders [58] and other harvestmen [6]. It is becoming clear that convergent evolution of troglomorphism within lineages is the expectation, rather than the exception. Consistent with this expectation, we have shown that troglomorphy has evolved independently at least three times within the *Sclerobunus* plus *Cyptobunus* complex, and perhaps as many as five times. This inference is founded on multigenic phylogenetics, explicit hypothesis testing, newly-developed relaxed clock methods, and quantitative consideration of morphological variation. Our perspective is unique in that we uncovered an apparently time-correlated dynamic, in an unexpected geographic region, in sometimes surprising microhabitats.

The sample available for this study almost certainly underestimates the actual number of independent transitions to troglomorphy, for several reasons. First, there are many known populations of cave-inhabiting troglomorphic *Sclerobunus* for which molecular phylogenetic data has yet to be gathered (e.g., Terrero Cave, Cement Creek Cave). Also, Briggs [20] described a troglomorphic juvenile ‘triaenonychid’ from a cave in southern Idaho that was placed near *Cyptobunus*, and Welbourn [59] reports populations of *Sclerobunus* from caves in southern Arizona, some well below the elevation at which surface *Sclerobunus* can be found. Finally, a likely majority of western caves have yet to be discovered and/or explored. Clearly, a full account of troglomorphic evolution in this group requires more field effort.

We may have also underestimated transitions to troglomorphy because *clades* of exclusively troglomorphic taxa (e.g., *Cyptobunus*, Cave of the Winds plus Mallory) could represent instances of *multiple* independent derivations. This is particularly likely for *Cyptobunus*, where currently described species and subspecies are found in geographically disjunct cave systems, separated by hundreds of kilometers of low elevation habitat that is obviously uninhabitable and lacks subterranean connectivity (see Fig. 1). *Cyptobunus* taxa are most likely independently derived from surface populations that have since gone extinct, leaving an array of extant, troglomorphic populations that share common ancestry, but not a single troglomorphic common ancestor.

### Time and Morphological Divergence

A logical expectation for cave-dwelling organisms is that the degree of troglomorphism is correlated with time, i.e., more highly-modified taxa are expected to be relatively old. While quantifying a “troglomorphy index” is reasonably straightforward, quantifying time available for evolution is more problematic, for several reasons. In the absence of molecular clock information, one might rely on cave ages to place upper limits on cave species ages, but studies have shown that troglomorphic species are sometimes older than the caves they inhabit [60,61; see below]. With molecular clock data, there is inherent error in time estimates themselves, but there are also potential problems with incomplete taxon/population sampling, and teasing apart cladogenic from post-speciation troglomorphic evolution [44], [62]. For example, a sparse phylogenetic sample of cave-dwellers, with missing surface taxa (either because of incomplete sampling or extinction), would be expected to overestimate the time available for troglomorphic evolution. One example of a study that assessed the relationship between time (based on geological evidence) and troglomorphy is that of Wessel et al., [60]; these authors found no correlation in cave planthoppers.

To investigate the troglomorphy/time relationship in sclerobunines, we used PC1 loadings as a proxy for troglomorphism. We correlated this variable with estimated mean time since divergence from *non-troglomorphic* sister taxa/populations (values from two-calibration BEAST analyses), and found a strong positive relationship (Table 3 and Fig. 3B). This correlation raises the interesting possibility of being able to predict taxon age from a simple troglomorphy index in this group. We have reasonable confidence in this relationship for three reasons. First, our sampling of known *extant* taxa and populations is relatively complete. We note that our sampling of surface taxa near *Cyptobunus* appears sparse (Fig. 2), but surface sclerobunines have in fact never been found in these geographic regions. Second, the observed correlation between troglomorphy and time does not depend upon *absolute* accuracy of time estimates, but rather only on the relative temporal ordering of divergence estimates. We have no basis to question the relative temporal ordering of these divergence estimates. Finally, a closer consideration of morphology shows a perhaps ordered evolution of troglomorphic characters, not captured by the simple morphological proxy. “Intermediate” forms (Fault and Skeleton Caves) show slight depigmentation and a small increase in leg length, whereas *Cyptobunus* show further appendage and spine attenuation. Eye reduction may be the last character affected, as this is not seen in *Cyptobunus*, but is seen in apparently more ancient eyeless travunioids (*Speleonychia* and *Speleomaster*; Fig. 5).

### Pleistocene Effects Model

A frequently-cited *temporal* model for the evolution of temperate cave-dwellers is the Pleistocene Effects model [63], [64]. Under this model, moist conditions of Pleistocene glacial periods allow connections to lower-elevation caves, while drier conditions of interglacial periods “strand” cryophilic populations underground, with subsequent evolution in isolated cave habitats.

In *Sclerobunus*, it appears that the evolution of “weakly” troglomorphic forms (i.e., with slightly reduced pigmentation, slight increase in leg length, no reduction in eye mound) is consistent with the Pleistocene Effects model. Three-calibration BEAST estimates for the divergence of Fault and Skeleton cave populations from geographically-proximate surface populations are Pleistocene in age (95% HPD 0.4-1.6, 0.7-1.2, respectively). In each case, the geographic setting is also consistent with a recent surface-to-cave climatic shift model. Fault Cave is located on a south-facing hillside at 1850 meters, surrounded by chaparral/grassland habitats (closest known surface population is 23 km distant, but likely closer), while Skeleton Cave is located only 200 meters upslope from a forested habitat (closest known surface population 3.5 km distant). These instances of cave and surface populations in close proximity invite population genetics studies, with larger genetic sample sizes and denser surface sampling closer to the caves. Such studies might be able to disentangle a recurrent gene flow model from shared ancestral genetic polymorphism, and allow more accurate estimates of divergence time [4], [5].

Absolute time estimates for the more highly troglomorphic sclerobunine lineages predate the Pleistocene, as has been found for other temperate cave-dwelling taxa [2], [45], [46]. Three-calibration estimates suggest Pliocene or Miocene divergences for *Cyptobunus* and Taos troglomorphs (Fig. 5), whereas two-calibration BEAST analyses suggest Miocene divergences for the common ancestor of the Cave of the Winds plus Mallory clade (Fig. S1). Other troglomorphic travunioids in our sample (*Speleonychia* and *Speleomaster*) also appear relatively ancient. We admit that our temporal analyses are hindered by a lack of additional calibration points, but this is a difficulty often faced in analyses of understudied arthropod groups with a sparse fossil record. We contend that our time estimates are reasonable, if not *underestimates*. Giribet et al., [29, fig. 10], using a relaxed clock analysis for multiple genes and multiple fossil calibration points, estimated a divergence value for the common ancestor of *Erebomaster* plus *Theromaster* at over 100 Ma. Our estimates for this same node are about four times shallower. We also note that estimated absolute rates of sclerobunine COI evolution, as implied by relaxed clock analyses, are consistent with arthropod rates reported in the literature (Table S6).

### Evolution of “Surface” Troglomorphs

At Taos, troglomorphic specimens were found in deep stony debris in a north-facing forest at an elevation of 2850 meters, syntopic with typical forms found under rocks and logs resting on the surface (Fig. 1). The troglomorphic forms inhabit a microhabitat classified as MSS [milieu souterrain superficial – superficial underground compartment; 65], which in montane southern Europe has been shown to house many populations and species of troglomorphic invertebrates [66], [67]. Such habitats are generally poorly-studied in North America. Interestingly, Peck [68] long-ago noted troglomorphic cave beetles from deep litter in high-elevation forests of central NM. Indeed, the taxonomic composition of these litter faunas reminded him so greatly of cave faunas that he referred to these as the “southwestern montane forest cave community”. Non-cave troglomorphs have actually been found in many arthropod taxa [23], [25], [69], [70], but their relationship to either surface or cave-dwelling forms has rarely been studied.

The syntopic surface and troglomorphic Taos forms are recovered as phylogenetic sister taxa, but are genetically divergent for all genes, differ by 8.7% for CO1 (uncorrected distance), and are estimated to have diverged in the Mio-Pliocene (95% HPD 2.9–8.2 Ma). Because the troglomorphs are extremely rare – we have visited this site on four separate occasions and have collected a total of 3 specimens (no adult males) – we are uncertain of both the full geographic distribution of this form, and the geographic context of divergence from surface ancestors (e.g., in allopatry versus parapatry). More work in western MSS habitats is needed, as the evolution of troglomorphism in these habitats greatly impacts interpretation of the evolution of cave forms. For example, it could be argued that troglomorphs originate in MSS habitats, with such forms secondarily invading cave habitats. An example might be the troglomorphs found in Cave of the Winds and Mallory caves, as both of these caves are found along the eastern edge of the Colorado Front Range, in potential contact with montane MSS. Under this model, cave-dwelling forms share a troglomorphic common ancestor, which evolved troglomorphism in a *non-cave* habitat. The highly-troglomorphic (and apparently ancient, Fig. 5) *Speleonychia*, found in Cascadian lava tubes dated at less than 1 Ma [71], is another possible example of this dynamic.

### Species Discovery and Conservation

We hypothesize that at least three new species have been discovered via our combination of fieldwork and systematic research. These include *S. r. idahoensis* from the Northern Rockies of Idaho, geographically-isolated from Cascadian *S. nondimorphicus* without known populations in intervening xeric habitats. Interestingly, these taxa are quite genetically divergent (7.63% average uncorrected pairwise divergence for CO1), but surprisingly similar in both somatic and penis morphology (Figs. 3 and 6). We hypothesize that Taos troglomorphic specimens represent a unique species, following the above-mentioned genetic and morphological divergence from syntopic forms. This species is currently only known from a single site, but likely has a larger geographic distribution. Finally, troglomorphic populations from Cave of the Winds and Mallory Cave likely represent one, or perhaps two, undescribed species. This hypothesis is based on observed divergence in somatic morphology, penis morphology, and consistent divergence for multiple genes. Shear et al., [13] recently reported on similar species discovery in cave-limited millipedes from the western US, and stressed the need for further cave exploration, integrative systematic research, and conservation attention. As noted by Shear et al., [13], climate change may ultimately dramatically impact these easily-perturbed ecosystems, and their many endemic, habitat-specialized inhabitants.

## Supporting Information

Figure S1 BEAST phylogram. Based on two calibration points (arrows), with 95% confidence intervals for all estimated nodes. Confidence intervals for the root of the tree and the outgroup clade are given below the nodes. Posterior probabilities of less than 95 are shown with red node bars. Troglomorphic populations/taxa indicated by special icons. Time for geologic epochs from the U.S. Geological Survey Geologic Names Committee, 2007, Divisions of geologic time-Major chronostratigraphic and geochronologic units: U.S. Geological Survey Fact Sheet 2007-3015, 2 p.
(0.62 MB EPS)

Table S1 Sample Information and GenBank numbers.
(0.16 MB DOC)

Table S2 Morphometric Measurements. Body and scute length measurements were taken dorsally at the midline, while length was taken at the widest point. Eye mound height was taken from the tallest point of the mound from the bottom of the eye and width was taken from the outside edge of both eyes at the widest point. Chelicerae length and width were taken at the longest points in a dorsal view for both segments. Palpal length and depth measurements were taken for each segment in lateral view except for the tarsal segment, which was taken in dorsal view. Leg length measurements were taken retrolaterally for the trochanter, femur, patella, tibia, and metatarsus and were measured between the most distant points on either side of the segment.
(0.30 MB DOC)

Table S3 PCR Primers.
(0.04 MB DOC)

Table S4 Model selection and testing results.
(0.03 MB DOC)

Table S5 Estimated divergence times from BEAST analyses (mean and confidence intervals).
(0.04 MB DOC)

Table S6 Estimated rates of COI evolution as implied by 2-calibration and 3-calibration BEAST analyses.
(0.04 MB DOC)

## References

[1] Culver DC, Kane TC, Fong DW (1995). Adaptation and Natural Selection in Caves.. The Evolution of Gammarus minus.

[2] Leys R, Watts CHS, Cooper SJB, Humphreys WF (2003). Evolution of subterranean diving beetles (Coleoptera: Dytiscidae: Hydroporini, Bidessini) in the arid zone of Australia.. Evolution.

[3] Wiens JJ, Chippindale PT, Hillis DM (2003). When are phylogenetic analyses misled by convergence? A case study in Texas cave salamanders.. Systematic Biology.

[4] Niemiller ML, Fitzpatrick BM, Miller BT (2008). Recent divergence with gene flow in Tennessee cave salamanders (Plethodontidae: *Gyrinophilus*) inferred from gene genealogies.. Molecular Ecology.

[5] Villacorta C, Jaume D, Oromi P, Juan C (2008). Under the volcano: phylogeography and evolution of the cave-dwelling *Palmorchestia hypogaea* (Amphipoda, Crustacea) at La Palma (Canary Islands).. BMC Biology.

[6] Hedin M, Thomas SM (2010). Molecular systematics of eastern North American Phalangodidae (Arachnida: Opiliones: Laniatores), demonstrating convergent morphological evolution in caves.. Molecular Phylogenetics and Evolution.

[7] Losos JB, Miles DB, Wainwright PC, Reilly SM (1994). Adaptation, constraint, and the comparative method: phylogenetic issues and methods.. Ecological Morphology: Integrative Organismal Biology.

[8] Christiansen KA (1962). Proposition pour la classification des animaux cavernicloes.. Spelunca.

[9] Culver DC, Master LL, Christman MC, Hobbs HH (2000). Obligate cave fauna of the 48 contiguous United States.. Conservation Biology.

[10] Culver DC, Christman MC, Elliott WR, Hobbs HH, Reddell JR (2003). The North American obligate cave fauna: regional patterns.. Biodiversity and Conservation.

[11] Peck SB (1980). Climatic change and the evolution of cave invertebrates in the Grand Canyon, Arizona.. National Speleological Society Bulletin.

[12] Peck SB (1981). The invertebrate fauna of the caves of the Uinta Mountains, northeastern Utah.. Great Basin Naturalist.

[13] Shear WA, Taylor SJ, Judson Wynne J, Krejca JK (2009). Cave millipeds of the United States. VIII. New genera and species of polydesmidan millipeds from caves in the southwestern United States (Diplopoda, Polydesmida, Macrosternodesmidae).. Zootaxa.

[14] Forster RR (1965). Harvestmen of the suborder Laniatores from New Zealand caves.. Records Otago Museum.

[15] Maury EA (1988). Triaenonychidae sudamericanos V. Un Nuevo genero de opiliones cavernicola del la Patagonia (Opiliones, Laniatores).. Mem de Biospelogie.

[16] Pérez González A, Kury AB (2002). A new remarkable troglomorphic Gonyleptid from Brazil Arachnida, Opiliones, Laniatores).. Revista Ibérica de Arachnologia.

[17] Karaman IM (2005). *Trojanella serbica gen. n., sp. n.*, a remarkable new troglobitic travunioid (Opiliones, Laniatores, Travunioidea).. Revue Suisse de Zoologie.

[18] Briggs TS (1971). The harvestmen of the family Triaenonychidae in North America.. Occasional Papers of the California Academy of Sciences.

[19] Briggs TS (1971). Relict harvestmen from the Pacific Northwest.. Pan-Pacific Entomologist.

[20] Briggs TS (1974). Troglobitic harvestmen recently discovered in North American lava tubes (Travuniidae, Erebomastridae, Triaenonychidae: Opiliones).. Journal of Arachnology.

[21] Cokendolpher JC (2004). Revalidation of the harvestman genus *Chinquipellobunus* (Opiliones: Stygnopsidae).. Texas Memorial Museum, Speleological Monographs.

[22] Ubick D, Briggs TS (1992). The harvestmen family Phalangodidae. 3. Revision of *Texella* (Opiliones: Laniatores).. Texas Memorial Museum, Speleological Monographs.

[23] Ubick D, Briggs TS (2002). The harvestmen family Phalangodidae 4. A review of the genus *Banksula* (Opiliones, Laniatores).. Journal of Arachnology.

[24] Ubick D, Briggs TS (2004). The harvestman family Phalangodidae. 5. New records and species of *Texella* Goodnight and Goodnight (Opiliones: Laniatores).. Texas Memorial Museum, Speleological Monographs.

[25] Ubick D, Briggs TS (2008). The harvestman family Phalangodidae 6. Revision of the *Sitalcina* complex (Opiliones, Laniatores).. Proceedings California Academy Sciences.

[26] Chambers SM (1993). Department of the Interior, Fish and Wildlife Service, 50 CFR Part 17, RIN 1018-AC06. Endangered and threatened wildlife and plants: Coffin Cave mold beetle (*Batrisodes texanus*) and the Bone Cave harvestmen (*Texella reyesi*) determined to be endangered.. Federal Register.

[27] Longacre C (2000). Department of the Interior, Fish and Wildlife Service, 50 CFR Part 17, RIN 1018-AF33. Final rule to list nine Bexar County, Texas invertebrate species as endangered.. Federal Register.

[28] Giribet G, Kury AB, Pinto-da-Rocha R, Machado G, Giribet G (2007). Chapter 3. Phylogeny and Biogeography.. Harvestmen: The Biology of Opiliones.

[29] Giribet G, Vogt L, Pérez González A, Sharma P, Kury AB (2009). A multilocus approach to harvestman (Arachnida: Opiliones) phylogeny with emphasis on biogeography and systematics of Laniatores.. Cladistics.

[30] Martens J (1986). Die grossgliederung der Opiliones und die evolution der ordung (Arachnida).. X International Congress of Arachnology.

[31] Derkarabetian S (2010). Phylogenetics of the Sclerobuninae (Arachnida: Opiliones: Laniatores): Systematics, Troglomorphic Evolution, and Phylogeography.. MS Thesis, San Diego State University.

[32] Thomas SM, Hedin M (2006). Natural history and distribution of the enigmatic southern Appalachian opilionid, *Fumontana deprehendor* Shear (Laniatores: Triaenonychidae), with an assessment of morphological variation.. Zootaxa.

[33] Ronquist F, Huelsenbeck JP, van der Mark P (2005). http://mrbayes.csit.fsu.edu/manual.php.

[34] Nylander JAA, Ronquist F, Huelsenbeck JP, Nieves-Aldre JL (2004). Bayesian phylogenetic analysis of combined data.. Systematic Biology.

[35] Kass RE, Raftery AE (1995). Bayes Factors.. Journal of the American Statistical Association.

[36] Thomas SM, Hedin M (2008). Multigenic phylogeographic divergence in the paleoendemic southern Appalachian opilionids *Fumontana deprehendor* Shear (Opiliones, Laniatores, Triaenonychidae).. Molecular Phylogenetics and Evolution.

[37] Hedin M, Derkarabetian S, McCormack M, Richart C, Shultz JW (2010). The phylogenetic utility of the nuclear protein-coding gene EF-1a for resolving recent divergences in Opiliones, emphasizing intron evolution.. Journal of Arachnology.

[38] Maddison DR, Maddison WP (2003). MacClade 4, Release Version 4.07..

[39] ThompsonJD, Gibson TJ, Plewniak F, Jeanmougin F, Higgins DG (1997). The ClustalX windows interface: flexible strategies for multiple sequence alignment aided by quality analysis tools.. Nucleic Acids Research.

[40] Huelsenbeck JP, Ronquist F (2001). MRBAYES: Bayesian inference of phylogeny, Version 3.0b4.. Bioinformatics.

[41] Nylander JA (2003). MrModeltest 1.1b..

[42] Drummond AJ, Ho S, Phillips M, Rambaut A (2006). Relaxed phylogenetics and dating with confidence.. PLoS Biology.

[43] Drummond AJ, Rambaut A (2007). BEAST: Bayesian evolutionary analysis by sampling trees.. BMC Evolutionary Biology.

[44] Porter ML, Dittmar K, Pérez-Losada M (2007). How long does evolution of the troglomorphic form take? Estimating divergence times in *Astyanax mexicanus*.. Acta Carsologica.

[45] Page TJ, Humphreys WF, Hughes JM (2008). Shrimps down under: Evolutionary relationships of subterranean crustacean from Western Australia (Decapoda: Atyidae: Stygiocaris).. PLoS ONE.

[46] Faille A, Ribera I, Deharveng L, Bourdeau C, Garnery L (2010). A molecular phylogeny shows the single origin of the Pyrenean subterranean Trechini ground beetles (Coleoptera: Carabidae).. Molecular Phylogenetics and Evolution.

[47] Eberhard WG (1985). Sexual selection and animal genitalia..

[48] Pinto-da-Rocha R, Giribet G, Pinto-da-Rocha R, Machado G, Giribet G (2007). Chapter 4, Taxonomy.. Harvestmen: The Biology of Opiliones.

[49] Brunsfeld SJ, Sullivan J, Soltis DE, Soltis PS, Silvertown J, Antonovics J (2001). Comparative phylogeography of northwestern North America: a Synthesis.. Integrating Ecology and Evolution in a Spatial Context.

[50] Carstens BC, Brunsfeld SJ, Demboski JR, Good JM, Sullivan J (2005). Investigating the evolutionary history of the Pacific Northwest mesic forest ecosystem: Hypothesis testing within a comparative phylogeographic framework.. Evolution.

[51] Ubick D, Dunlop J (2005). On the placement of the Baltic amber harvestman *Gonyleptes nemastomoides* Koch and Berendt, 1854, with notes on the phylogeny of Cladonychiidae (Opiliones, Laniatores, Travunioidea).. Mitteilungen aus dem Museum für Naturkunde in Berlin – Geowissenschaftliche Reihe.

[52] White WB (1988). Geomorphology and hydrology of karst terrains..

[53] Moore GW, Sullivan N (1997). Speleology – Caves and the cave environment: Third edition..

[54] Luiszer FG (2007). Timing of passage development and sedimentation at Cave of the Winds, Manitou Springs, Colorado, USA.. Acta Carsologica.

[55] Shultz JW, Pinto-da-Rocha P, Pinto-da-Rocha R, Machado G, Giribet G (2007). Chapter 2. Morphology and Functional Anatomy.. Harvestmen: The Biology of Opiliones.

[56] Dowling TE, Martasian DP, Jeffrey WR (2002). Evidence for multiple genetic forms with similar eyeless phenotypes in the blind cavefish, *Astyanax mexicanus*.. Molecular Biology and Evolution.

[57] Wilcox TP, García de León FJ, Hendrickson DA, Hillis DM (2004). Convergence among cave catfishes: long-branch attraction and a Bayesian relative rates test.. Molecular Phylogenetics and Evolution.

[58] Arnedo MA, Múrria C, Macías-Hernández N, Oromí P, Ribera C (2007). The dark side of an island radiation: Systematics and evolutionary patterns of troglobitic spiders of the genus Dysdera (Araneae, Dysderidae) in the Canary Islands.. Invertebrate Systematics.

[59] Welbourn WC (1999). Invertebrate cave fauna of Kartchner Caverns, Kartchner Caverns, Arizona.. Journal of Cave and Karst Studies.

[60] Wessel A, Erbe P, Hoch H (2007). Pattern and process: Evolution of troglomorphy in the cave-planthopper of Australia and Hawaii – preliminary observations (Insecta: Hemiptera: Fulgoromorpha: Cixiidae).. Acta Carsologica.

[61] Hoch H, Howarth FG (1989). Six new cavernicolous cixiid planthoppers in the genus *Solonaima* from Australia (Homoptera: Fulgoroidea).. Systematic Entomology.

[62] Porter ML (2007). Subterranean biogeography: what have we learned from molecular techniques?. Journal of Cave and Karst Studies.

[63] Barr TC, Dobzhansky T, Hecht MK, Steere WC (1968). Cave ecology and the evolution of troglobites.. Evolutionary Biology.

[64] Barr TC, Holsinger JR (1985). Speciation in cave faunas.. Annual Review of Ecology and Systematics.

[65] Juberthie C, Delay B (1981). Ecological and biological implications of a “superficial underground compartment”.. Proceedings of the International Congress of Speleology.

[66] Ruzicka J (1998). Cave and rock debris dwelling species of the *Choleva agilis* species group from central Europe (Coleoptera, Leiodidae: Cholevinae).. Regional Museum of Natural Science, Torino.

[67] Ruzicka J (1999). The first steps in subterranean evolution of spiders (Araneae) in central Europe.. Journal of Natural History.

[68] Peck SB (1978). New montane *Ptomaphagus* beetles from New Mexico and zoogeography of southwestern caves (Coleoptera, Leiodidae, Catopinae).. Southwestern Naturalist.

[69] Howarth FG (1983). Ecology of cave arthropods.. Annual Review of Entomology.

[70] Oromi P, Medina AL, Tejedor ML (1986). On the existence of a superficial underground compartment in the Canary Islands.. Congreso Internacional de Espeleologia.

[71] Greeley R, Hyde JH (1972). Lava tubes of the cave basalt, Mount St. Helens, Washington.. Geological Society of America Bulletin.

