# Supplementary material for: Repeated and Time-Correlated Morphological Convergence in Cave-Dwelling Harvestmen (Opiliones, Laniatores) from Montane Western North America
Source: PLoS One. 2010 May 7;5(5):e10388. doi: 10.1371/journal.pone.0010388 (PMC2866537; doi:10.1371/journal.pone.0010388)
Supplement: Table S1 — Sample Information and GenBank numbers. (0.16 MB DOC) [file pone.0010388.s002.doc]

**Table S1 – Sample Information and GenBank numbers**

| **Taxon** | **Voucher Number** | **Locality Number** | **Locality information** | **Lat, Long** | **CO1 GenBank** | **28S GenBank** | **EF1a GenBank** |
| --- | --- | --- | --- | --- | --- | --- | --- |
|  |  |  |  |  |  |  |  |
| **TRAVUNIODEA** |  |  |  |  |  |  |  |
| **Cladonychiidae** |  |  |  |  |  |  |  |
| *Erebomaster flavescens* | OP522 |  | VA: Washington County, Brumley Creek | 47.4339,  -121.7765 | HM056722 | HM056639 | -- |
| *Theromaster sp.* | OP359 |  | VA: Scott County, Cliff Mtn. | 36.7494,  -82.7787 | HM056723 | HM056640 | -- |
| *Cryptomaster leviathan* | OP1641 |  | OR: Lane County, Clark Creek Campground | 43.9733,  -122.5808 | HM056724 | HM056641 | -- |
| *Speleomaster lexi* | OP1691 |  | ID: Lincoln County, T Cave (Tee Cave) | Withheld | HM056725 | HM056642 | -- |
|  |  |  |  |  |  |  |  |
| **Briggsidae** |  |  |  |  |  |  |  |
| *Briggsus flavescens* | OP1052 |  | OR: Columbia County, southwest of Clatskanie | 46.0632,  -123.2675 | HM056726 | HM056643 | -- |
|  |  |  |  |  |  |  |  |
| **Travuniidae** |  |  |  |  |  |  |  |
| *Speleonychia sengeri* | OP1683 |  | WA: Skamania County, Big Cave | Withheld | HM056727 | HM056644 | -- |
|  |  |  |  |  |  |  |  |
| **North American ‘Triaenonychids’** |  |  |  |  |  |  |  |
| **Paranonychinae** |  |  |  |  |  |  |  |
| *Paranonychus brunneus* | OP1053 |  | OR: Multnomah County, Tanner Creek Road | 45.6345,  -121.9438 | HM056728 | HM056645 | HM056691 |
| *Paranonychus brunneus* | OP1671 |  | WA: Jefferson County, Ruby Beach | 47.7098,  -124.4137 | HM056729 | HM056646 | -- |
| *Paranonychus brunneus* | OP1784 |  | OR: Clatsop County, Saddle Mountain SP | 45.9629,  -123.6900 | HM056730 | HM056647 | -- |
| *Metanonychus setulus* | OP974 |  | CA: Del Norte County, Del Norte Coast Redwood SP | 41.6954,  -124.1191 | HM056731 | HM056648 | -- |
| *Metanonychus setulus* | OP985 |  | OR: Josephine County, Grave Creek | 42.6414,  -123.5062 | HM056732 | HM056649 | -- |
| *Metanonychus setulus* | OP1966 |  | OR: Lane County, Williamette NF | 43.9725,  -122.5793 | HM056733 | HM056650 | -- |
|  |  |  |  |  |  |  |  |
| **Sclerobuninae** |  |  |  |  |  |  |  |
| *Z. acuta* | OP748, OP750 |  | CA: San Mateo County, Pescadero Road | 37.2998,  -122.2696 | HM056734 (OP748) | HM056651 (OP748) | HM056692 (OP750) |
| *Z. tioga* | OP2445 |  | CA: Tuolumne County, near Yosemite NP | 37.7599,  -119.8452 | HM056735 | HM056652 | -- |
|  |  |  |  |  |  |  |  |
| *C. cavicolens* | OP2143, OP2144 |  | MT: Jefferson County, Lewis and Clark Caverns | 45.8386,  -111.8668 | GQ870664 HM056736 | HM056653 HM056654 | GQ872179 HM056693 |
| *C. u. madhousensis* | OP239 |  | UT: Utah County, Professor Buss Cave | Withheld | HM056737 | HM056655 | HM056694 |
| *C. u. ungulatus* | OP1229 |  | NV: White Pine County, Great Basin NP, Ice Cave | Withheld | HM056738 | HM056656 | HM056695 |
| *C. u. ungulatus* | OP1232 |  | NV: White Pine County, Great Basin NP, Model Cave | Withheld | HM056739 | HM056657 | HM056696 |
|  |  |  |  |  |  |  |  |
| *S. nondimorphicus* | OP222 |  | WA: Lewis County, Gifford Pinchot SP | 46.5399,  -121.7519 | GQ200413 | HM056658 | HM056697 |
| *S. nondimorphicus* | OP1056 |  | OR: Clatsop County, Ecola SP | 45.9221,  -123.9767 | GQ870663 | HM056659 | GQ872178 |
| *S. r. idahoensis* | OP1635 |  | ID: Latah County, Benewah Co. Line | 47.0377,  -116.6735 | HM056740 | HM056660 | HM056698 |
| *S. r. idahoensis* | OP1649 |  | ID: Shoshone County, Hobo Cedar Grove | 47.086,  -116.1128 | HM056741 | HM056661 | HM056699 |
|  |  |  |  |  |  |  |  |
| *S. r. robustus* | OP2568 | 1 | CO: Boulder County, Mallory Cave | Withheld | HM056742 | HM056662 | HM056700 |
| *S. r. robustus* | OP1127 | 2 | CO: El Paso County, Cave of the Winds | 38.8728,  -104.9201 | HM056743 | HM056663 | HM056701 |
| *S. r. robustus* | OP2686 | 3 | CO: Gilpin Co., Apex Valley Road | 39.8192,  -105.5132 | HM056744 | HM056664 | HM056702 |
| *S. r. robustus* | OP2569 | 4 | CO: Jefferson County, Fault Cave | Withheld | HM056745 | HM056665 | -- |
| *S. r. robustus* | OP2679 | 5 | CO: Garfield Co., Hanging Lake Trail | 39.5985,  -107.191 | HM056746 | HM056666 | HM056703 |
| *S. r. robustus* | OP2567 | 6 | CO: Garfield County, Skeleton Cave | Withheld | HM056747 | HM056667 | -- |
| *S. r. robustus* | OP1140 | 7 | CO: Lake County, NE of Leadville | 39.3415,  -106.2229 | HM056748 | HM056668 | HM056704 |
| *S. r. robustus* | OP1215 | 8 | CO: Chaffee County, near Garfield | 38.5494,  -106.2557 | HM056749 | HM056669 | HM056705 |
| *S. r. robustus* | OP1122 | 9 | CO: Gunnison County, south of Gothic | 38.9397,  -106.4072 | GQ870667 | HM056670 | GQ872182 |
| *S. r. robustus* | OP1164 | 10 | CO: Custer County, Wet Mountains | 38.1335,  -105.1791 | GQ870668 | HM056671 | GQ872183 |
| *S. r. robustus* | OP2109 | 11 | CO: Mineral County, south of Creede | 37.8139,  -106.9147 | HM056750 | HM056672 | HM056706 |
| *S. r. robustus* | OP1210 | 12 | CO: Rio Grande County, Church Creek Trailhead | 37.6481,  -106.652 | HM056751 | HM056673 | HM056707 |
| *S. r. robustus* | OP1223 | 13 | CO: La Plata County, Haviland Lake | 37.5329,  -107.807 | HM056752 | HM056674 | HM056708 |
| *S. r. robustus* | OP2115 | 14 | NM: Rio Arriba County, north of Chama | 36.9394,  -106.5493 | HM056753 | HM056675 | HM056709 |
| *S. r. robustus* | OP885 | 15 | NM: Sandoval County, Jemez Mountains | 35.8384,  -106.4044 | GQ870665 | HM056676 | GQ872180 |
| *S. r. robustus* | OP893 | 16 | NM: Lincoln County, road to Ski Apache | 33.3969,  -105.7826 | HM056754 | HM056677 | -- |
| *S. r. robustus* | OP956 | 17 | NM: Otera County, Bradford Canyon | 32.978,  -105.7087 | HM056755 | HM056678 | HM056710 |
| *S. r. robustus* | OP1039 | 18 | AZ: Cochise County, Chiricahua Mountains | 31.9179,  -109.2725 | HM056756 | HM056679 | HM056711 |
| *S. r. robustus* | OP951 | 19 | NM: Grant County, Pinos Altos Mountains | 32.9287,  -108.1662 | HM056757 | HM056680 | HM056712 |
| *S. r. robustus* | OP905 | 20 | AZ: Apache County, Alpine Divide | 33.8936,  -109.1519 | HM056758 | HM056681 | HM056713 |
| *S. r. robustus* | OP964 | 21 | AZ: Pima County, Santa Catalina Mountains | 32.4265  -110.7424 | HM056759 | HM056682 | HM056714 |
| *S. r. robustus* | OP913 | 22 | AZ: Coconino County, San Francisco Mountains | 35.3289,  -111.7121 | HM056760 | HM056683 | HM056715 |
| *S. r. robustus* | OP1130 | 23 | UT: San Juan County, Abajo Mountains | 37.8763,  -109.4458 | HM056761 | HM056684 | HM056716 |
| *S. r. robustus* | OP1203 | 24 | UT: San Juan County, La Sal Mountains | 38.4154,  -109.2241 | HM056762 | HM056685 | HM056717 |
| *S. r. robustus* | OP1149 | 25 | CO: Dolores County, along Dolores River | 37.7679,  -107.9871 | GQ870666 | HM056686 | GQ872181 |
| *S. r. robustus* | OP2140 | -- | CO: Gunnison County, Cement Creek Cave #1 | Withheld | -- | -- | -- |
|  |  |  |  |  |  |  |  |
| *S. r. glorietus* | OP882, OP972 | 26 | NM: Taos County, Taos Ski Valley | 36.5976,  -105.4576 | HM056763, HM056764 | HM056687, HM056688 | HM056718, HM056719 |
| *S. r. glorietus* | OP890 | 27 | NM: Santa Fe County, Glorieta Canyon | 35.6118,  -105.7703 | HM056765 | HM056689 | HM056720 |
| *S. r. glorietus* | OP907 | 28 | NM: Torrance County, Manzano Mountains | 34.6658,  -106.3907 | HM056766 | HM056690 | HM056721 |
| *S. r. glorietus?* | CAS | -- | NM: Santa Fe County, Terrero Cave (CAS) | Withheld | -- | -- | -- |
