# Supplementary material for: Repeated and Time-Correlated Morphological Convergence in Cave-Dwelling Harvestmen (Opiliones, Laniatores) from Montane Western North America
Source: PLoS One. 2010 May 7;5(5):e10388. doi: 10.1371/journal.pone.0010388 (PMC2866537; doi:10.1371/journal.pone.0010388)
Supplement: Table S2 — Morphometric Measurements. Body and scute length measurements were taken dorsally at the midline, while length was taken at the widest point. Eye mound height was taken from the tallest point of the mound from the bottom of the eye and width was taken from the outside edge of both eyes at the widest point. Chelicerae length and width were taken at the longest points in a dorsal view for both segments. Palpal length and depth measurements were taken for each segment in lateral view except for the tarsal segment, which was taken in dorsal view. Leg length measurements were taken retrolaterally for the trochanter, femur, patella, tibia, and metatarsus and were measured between the most distant points on either side of the segment. (0.30 MB DOC) [file pone.0010388.s003.doc]

| **Male # / Loc #** |  | **Variable** | **1** | **2** | **3** | **4** | **5** | **6** | **7** | **8** | **9** |
| --- | --- | --- | --- | --- | --- | --- | --- | --- | --- | --- | --- |
|  | **Species/Genetic Clade** |  | **BodyL** | **ScuL** | **ScuW** | **ETubH** | **ETubW** | **Chel1L** | **Chel1W** | **Chel2L** | **Chel2W** |
| 26 | *S. nondimorphicus* |  | 3.125 | 2.328 | 2.313 | 0.389 | 0.188 | 0.511 | 0.294 | 0.911 | 0.311 |
| 30 | *S. nondmorphicus* |  | 2.891 | 2.344 | 2.475 | 0.389 | 0.188 | 0.544 | 0.294 | 0.889 | 0.311 |
| 28 | *S. r. idahoensis* |  | 2.781 | 2.375 | 2.531 | 0.389 | 0.184 | 0.483 | 0.289 | 0.844 | 0.311 |
| 29 | *S. r. idahoensis* |  | 2.694 | 2.272 | 2.469 | 0.379 | 0.18 | 0.411 | 0.283 | 0.794 | 0.317 |
| 9 / 26 | *S. r. glorietus* |  | 2.063 | 1.688 | 1.8 | 0.244 | 0.094 | 0.344 | 0.206 | 0.633 | 0.228 |
| 19 / 26 | *S. r. glorietus* |  | 2.17 | 1.808 | 1.788 | 0.26 | 0.09 | 0.397 | 0.218 | 0.653 | 0.244 |
| 20 / 26 | *S. r. glorietus* |  | 2.075 | 1.705 | 1.72 | 0.222 | 0.11 | 0.378 | 0.206 | 0.601 | 0.233 |
| 12 / 21 | *S. r. robustus* |  | 2.734 | 2.625 | 2.703 | 0.339 | 0.175 | 0.689 | 0.322 | 0.984 | 0.344 |
| 13 / 21 | *S. r. robustus* |  | 3 | 2.5 | 2.516 | 0.367 | 0.171 | 0.667 | 0.322 | 0.933 | 0.339 |
| 14 / 21 | *S. r. robustus* |  | 2.75 | 2.594 | 2.719 | 0.328 | 0.135 | 0.644 | 0.311 | 0.917 | 0.35 |
| 1 / 12 | *S. r. robustus* |  | 2.53 | 2.34 | 2.46 | 0.33 | 0.125 | 0.55 | 0.27 | 0.857 | 0.27 |
| 2 / 12 | *S. r. robustus* |  | 2.73 | 2.338 | 2.328 | 0.33 | 0.1423 | 0.51 | 0.22 | 0.866 | 0.305 |
| 15 / 17 | *S. r. robustus* |  | 2.438 | 2.078 | 2.03 | 0.291 | 0.116 | 0.506 | 0.261 | 0.75 | 0.283 |
| 16 / 17 | *S. r. robustus* |  | 2.438 | 1.913 | 1.889 | 0.287 | 0.151 | 0.511 | 0.253 | 0.697 | 0.276 |
| 32 / 3 | *S. r. robustus* |  | 3.18 | 2.42 | 2.453 | 0.311 | 0.155 | 0.572 | 0.3 | 0.817 | 0.317 |
| 33 / 3 | *S. r. robustus* |  | 3 | 2.344 | 2.344 | 0.317 | 0.138 | 0.611 | 0.311 | 0.828 | 0.322 |
| 34 / 5 | *S. r. robustus* |  | 2.813 | 2.222 | 2.375 | 0.272 | 0.124 | 0.522 | 0.269 | 0.772 | 0.289 |
| 35 / 5 | *S. r. robustus* |  | 2.494 | 2 | 2.047 | 0.267 | 0.16 | 0.528 | 0.267 | 0.767 | 0.267 |
| 5 / 4 | *S. r. robustus* |  | 3.04 | 2.4375 | 2.48 | 0.254 | 0.13 | 0.556 | 0.278 | 0.875 | 0.315 |
| 25 | *S. r. robustus* |  | 2.563 | 2.059 | 2.2 | 0.247 | 0.119 | 0.459 | 0.249 | 0.738 | 0.256 |
| 8 | *S. r. glorietus* |  | 2.113 | 2 | 1.875 | 0.233 | 0.094 | 0.4 | 0.211 | 0.746 | 0.238 |
| 31 / 1 | *Sclerobunus sp* |  | 2.625 | 2.381 | 2.641 | 0.311 | 0.125 | 0.544 | 0.3 | 0.967 | 0.322 |
| 6 / 2 | *Sclerobunus sp* |  | 2.2 | 1.925 | 2.1 | 0.256 | 0.071 | 0.589 | 0.262 | 0.889 | 0.286 |
| 7 / 2 | *Sclerobunus sp* |  | 2.163 | 1.938 | 2.088 | 0.267 | 0.103 | 0.563 | 0.254 | 0.897 | 0.27 |
| 21 / 2 | *Sclerobunus sp* |  | 2.25 | 1.963 | 2.154 | 0.278 | 0.11 | 0.633 | 0.256 | 0.867 | 0.3 |
| 3 | *C. cavicolens* |  | 2.362 | 1.675 | 1.65 | 0.227 | 0.079 | 0.455 | 0.211 | 0.7 | 0.151 |
| 4 | *C. cavicolens* |  | 1.675 | 1.575 | 1.763 | 0.233 | 0.077 | 0.417 | 0.206 | 0.713 | 0.222 |
| 24 | *C. cavicolens* |  | 2.413 | 1.638 | 1.658 | 0.224 | 0.078 | 0.417 | 0.211 | 0.672 | 0.211 |
| 17 | *C. u. madhousensis* |  | 2.163 | 1.938 | 1.98 | 0.223 | 0.116 | 0.483 | 0.239 | 0.817 | 0.251 |
| 18 | *C. u. madhousensis* |  | 2.46 | 1.913 | 1.92 | 0.224 | 0.078 | 0.467 | 0.228 | 0.75 | 0.244 |
| 10 | *C. u. ungulatus* |  | 2.75 | 2.172 | 2.175 | 0.272 | 0.087 | 0.528 | 0.283 | 0.9375 | 0.228 |
| 11 | *C. u. ungulatus* |  | 2.781 | 2.172 | 2.15 | 0.288 | 0.083 | 0.516 | 0.278 | 0.944 | 0.228 |
| 22 | *C. u. ungulatus* |  | 3.172 | 2.152 | 2.27 | 0.28 | 0.087 | 0.605 | 0.278 | 0.906 | 0.3 |

| **Male # / Loc #** |  | **Variable** | **10** | **11** | **12** | **13** | **14** | **15** | **16** | **17** | **18** |
| --- | --- | --- | --- | --- | --- | --- | --- | --- | --- | --- | --- |
|  | **Species/Genetic Clade** |  | **PalpFL** | **PalpFD** | **PalpPL** | **PalpPD** | **PalpTL** | **PalpTD** | **PalpTL** | **PalpTW** | **L1Troch** |
| 26 | *S. nondimorphicus* |  | 1.238 | 0.548 | 0.744 | 0.4 | 0.906 | 0.433 | 0.811 | 0.322 | 0.433 |
| 30 | *S. nondmorphicus* |  | 1.238 | 0.533 | 0.733 | 0.389 | 0.944 | 0.439 | 0.828 | 0.294 | 0.45 |
| 28 | *S. r. idahoensis* |  | 1.19 | 0.548 | 0.709 | 0.394 | 0.889 | 0.476 | 0.77 | 0.305 | 0.45 |
| 29 | *S. r. idahoensis* |  | 1.051 | 0.555 | 0.733 | 0.383 | 0.817 | 0.448 | 0.743 | 0.269 | 0.394 |
| 9 / 26 | *S. r. glorietus* |  | 0.706 | 0.367 | 0.406 | 0.25 | 0.478 | 0.278 | 0.433 | 0.2 | 0.278 |
| 19 / 26 | *S. r. glorietus* |  | 0.744 | 0.38 | 0.444 | 0.25 | 0.567 | 0.293 | 0.5167 | 0.2 | 0.29 |
| 20 / 26 | *S. r. glorietus* |  | 0.698 | 0.372 | 0.417 | 0.244 | 0.513 | 0.294 | 0.45 | 0.189 | 0.294 |
| 12 / 21 | *S. r. robustus* |  | 1.22 | 0.811 | 0.772 | 0.472 | 0.944 | 0.522 | 0.739 | 0.361 | 0.428 |
| 13 / 21 | *S. r. robustus* |  | 1.366 | 0.817 | 0.778 | 0.483 | 0.952 | 0.533 | 0.722 | 0.333 | 0.456 |
| 14 / 21 | *S. r. robustus* |  | 1.254 | 0.802 | 0.756 | 0.45 | 0.937 | 0.5 | 0.772 | 0.328 | 0.428 |
| 1 / 12 | *S. r. robustus* |  | 1.15 | 0.7 | 0.66 | 0.4 | 0.86 | 0.44 | 0.67 | 0.277 | 0.4 |
| 2 / 12 | *S. r. robustus* |  | 1.11 | 0.67 | 0.672 | 0.377 | 0.809 | 0.4 | 0.683 | 0.277 | 0.394 |
| 15 / 17 | *S. r. robustus* |  | 0.978 | 0.6 | 0.544 | 0.372 | 0.67 | 0.367 | 0.583 | 0.258 | 0.35 |
| 16 / 17 | *S. r. robustus* |  | 0.897 | 0.54 | 0.53 | 0.339 | 0.65 | 0.358 | 0.55 | 0.233 | 0.313 |
| 32 / 3 | *S. r. robustus* |  | 1.206 | 0.649 | 0.713 | 0.394 | 0.872 | 0.422 | 0.75 | 0.267 | 0.433 |
| 33 / 3 | *S. r. robustus* |  | 1.175 | 0.683 | 0.733 | 0.402 | 0.889 | 0.45 | 0.772 | 0.294 | 0.422 |
| 34 / 5 | *S. r. robustus* |  | 1.111 | 0.613 | 0.639 | 0.356 | 0.8 | 0.378 | 0.7 | 0.256 | 0.433 |
| 35 / 5 | *S. r. robustus* |  | 1.05 | 0.53 | 0.622 | 0.324 | 0.79 | 0.356 | 0.706 | 0.244 | 0.399 |
| 5 / 4 | *S. r. robustus* |  | 1.26 | 0.63 | 0.72 | 0.4 | 0.92 | 0.4 | 0.82 | 0.28 | 0.45 |
| 25 | *S. r. robustus* |  | 1.094 | 0.533 | 0.633 | 0.336 | 0.772 | 0.356 | 0.696 | 0.223 | 0.383 |
| 8 | *S. r. glorietus* |  | 1.048 | 0.421 | 0.603 | 0.31 | 0.739 | 0.294 | 0.667 | 0.217 | 0.311 |
| 31 / 1 | *Sclerobunus sp* |  | 1.524 | 0.683 | 0.861 | 0.433 | 1.27 | 0.462 | 1.033 | 0.289 | 0.501 |
| 6 / 2 | *Sclerobunus sp* |  | 1.3 | 0.51 | 0.738 | 0.325 | 0.984 | 0.357 | 0.87 | 0.206 | 0.406 |
| 7 / 2 | *Sclerobunus sp* |  | 1.14 | 0.53 | 0.73 | 0.34 | 1.02 | 0.344 | 0.838 | 0.239 | 0.406 |
| 21 / 2 | *Sclerobunus sp* |  | 1.325 | 0.524 | 0.738 | 0.33 | 0.989 | 0.36 | 0.867 | 0.239 | 0.422 |
| 3 | *C. cavicolens* |  | 0.96 | 0.309 | 0.572 | 0.244 | 0.811 | 0.244 | 0.68 | 0.188 | 0.328 |
| 4 | *C. cavicolens* |  | 0.984 | 0.31 | 0.501 | 0.256 | 0.762 | 0.238 | 0.667 | 0.19 | 0.322 |
| 24 | *C. cavicolens* |  | 0.95 | 0.3 | 0.573 | 0.247 | 0.772 | 0.236 | 0.711 | 0.17 | 0.333 |
| 17 | *C. u. madhousensis* |  | 1.257 | 0.381 | 0.722 | 0.3 | 0.989 | 0.294 | 0.878 | 0.222 | 0.422 |
| 18 | *C. u. madhousensis* |  | 1.238 | 0.365 | 0.738 | 0.289 | 1 | 0.29 | 0.867 | 0.2 | 0.367 |
| 10 | *C. u. ungulatus* |  | 1.313 | 0.413 | 0.888 | 0.325 | 1.087 | 0.341 | 0.968 | 0.239 | 0.489 |
| 11 | *C. u. ungulatus* |  | 1.34 | 0.428 | 0.867 | 0.344 | 1.11 | 0.344 | 0.976 | 0.239 | 0.489 |
| 22 | *C. u. ungulatus* |  | 1.316 | 0.428 | 0.767 | 0.344 | 1.067 | 0.322 | 0.944 | 0.239 | 0.462 |

| **Male # / Loc #** |  | **Variable** | **19** | **20** | **21** | **22** | **23** | **24** | **25** | **26** | **27** |
| --- | --- | --- | --- | --- | --- | --- | --- | --- | --- | --- | --- |
|  | **Taxon** |  | **L1Fem** | **L1Pat** | **L1Tib** | **L1Met** | **L2Troch** | **L2Fem** | **L2Pat** | **L2Tib** | **L2Met** |
| 26 | *S. nondimorphicus* |  | 1.484 | 0.656 | 1.024 | 1.262 | 0.472 | 2.425 | 0.828 | 1.955 | 2.138 |
| 30 | *S. nondmorphicus* |  | 1.43 | 0.7 | 1.095 | 1.365 | 0.489 | 2.363 | 0.822 | 2.025 | 2.175 |
| 28 | *S. r. idahoensis* |  | 1.33 | 0.667 | 0.974 | 1.206 | 0.5 | 2.083 | 0.756 | 1.77 | 2.063 |
| 29 | *S. r. idahoensis* |  | 1.246 | 0.617 | 0.944 | 1.19 | 0.476 | 2.01 | 0.744 | 1.68 | 1.94 |
| 9 / 26 | *S. r. glorietus* |  | 0.75 | 0.411 | 0.606 | 0.667 | 0.306 | 1.083 | 0.467 | 0.961 | 0.989 |
| 19 / 26 | *S. r. glorietus* |  | 0.817 | 0.411 | 0.65 | 0.736 | 0.333 | 1.21 | 0.5 | 1.033 | 1.117 |
| 20 / 26 | *S. r. glorietus* |  | 0.761 | 0.4 | 0.611 | 0.683 | 0.306 | 1.087 | 0.469 | 0.956 | 1.033 |
| 12 / 21 | *S. r. robustus* |  | 1.254 | 0.639 | 0.961 | 1.151 | 0.478 | 1.816 | 0.722 | 1.571 | 1.8 |
| 13 / 21 | *S. r. robustus* |  | 1.222 | 0.633 | 0.928 | 1.103 | 0.478 | 1.72 | 0.733 | 1.54 | 1.684 |
| 14 / 21 | *S. r. robustus* |  | 1.22 | 0.611 | 0.944 | 1.2 | 0.517 | 1.77 | 0.746 | 1.571 | 1.784 |
| 1 / 12 | *S. r. robustus* |  | 1.175 | 0.525 | 0.867 | 1.19 | 0.411 | 1.675 | 0.69 | 1.47 | 1.68 |
| 2 / 12 | *S. r. robustus* |  | 1.254 | 0.619 | 0.921 | 1.167 | 0.428 | 1.75 | 0.64 | 1.53 | 1.68 |
| 15 / 17 | *S. r. robustus* |  | 0.961 | 0.489 | 0.75 | 0.881 | 0.369 | 1.389 | 0.589 | 1.23 | 1.384 |
| 16 / 17 | *S. r. robustus* |  | 0.908 | 0.483 | 0.717 | 0.822 | 0.372 | 1.314 | 0.558 | 1.175 | 1.278 |
| 32 / 3 | *S. r. robustus* |  | 1.305 | 0.594 | 0.989 | 1.238 | 0.467 | 1.856 | 0.682 | 1.65 | 1.84 |
| 33 / 3 | *S. r. robustus* |  | 1.254 | 0.6 | 0.967 | 1.22 | 0.456 | 1.86 | 0.667 | 1.62 | 1.88 |
| 34 / 5 | *S. r. robustus* |  | 1.278 | 0.578 | 0.958 | 1.214 | 0.467 | 1.9 | 0.678 | 1.68 | 1.86 |
| 35 / 5 | *S. r. robustus* |  | 1.254 | 0.55 | 0.922 | 1.175 | 0.45 | 1.87 | 0.65 | 1.62 | 1.869 |
| 5 / 4 | *S. r. robustus* |  | 1.41 | 0.62 | 1.05 | 1.36 | 0.489 | 2.14 | 0.72 | 1.788 | 2.088 |
| 25 | *S. r. robustus* |  | 1.197 | 0.581 | 0.876 | 1.062 | 0.416 | 1.72 | 0.663 | 1.53 | 1.68 |
| 8 | *S. r. glorietus* |  | 1.381 | 0.556 | 1.008 | 1.214 | 0.4 | 2.338 | 0.714 | 1.87 | 1.96 |
| 31 / 1 | *Sclerobunus sp* |  | 1.89 | 0.803 | 1.484 | 1.824 | 0.533 | 2.94 | 1.025 | 2.64 | 3 |
| 6 / 2 | *Sclerobunus sp* |  | 1.64 | 0.698 | 1.214 | 1.63 | 0.444 | 2.625 | 0.841 | 2.25 | 2.656 |
| 7 / 2 | *Sclerobunus sp* |  | 1.425 | 0.678 | 1.183 | 1.54 | 0.456 | 2.453 | 0.813 | 2.15 | 2.469 |
| 21 / 2 | *Sclerobunus sp* |  | 1.65 | 0.664 | 1.241 | 1.71 | 0.467 | 2.656 | 0.833 | 2.342 | 2.741 |
| 3 | *C. cavicolens* |  | 1.317 | 0.516 | 1.028 | 1.246 | 0.339 | 2.1 | 0.633 | 1.82 | 1.875 |
| 4 | *C. cavicolens* |  | 1.302 | 0.571 | 1.024 | 1.278 | 0.35 | 2.088 | 0.635 | 1.788 | 1.94 |
| 24 | *C. cavicolens* |  | 1.333 | 0.54 | 1.017 | 1.294 | 0.35 | 2.075 | 0.639 | 1.36 | 1.91 |
| 17 | *C. u. madhousensis* |  | 1.68 | 0.644 | 1.349 | 1.604 | 0.417 | 2.656 | 0.772 | 2.37 | 2.203 |
| 18 | *C. u. madhousensis* |  | 1.7 | 0.683 | 1.324 | 1.62 | 0.433 | 2.797 | 0.786 | 2.438 | 2.563 |
| 10 | *C. u. ungulatus* |  | 2.163 | 0.77 | 1.65 | 2.15 | 0.522 | 3.48 | 0.938 | 3.031 | 3.2 |
| 11 | *C. u. ungulatus* |  | 2.138 | 0.733 | 1.66 | 2.1 | 0.539 | 3.52 | 0.944 | 3.063 | 3.289 |
| 22 | *C. u. ungulatus* |  | 2.063 | 0.733 | 1.606 | 1.944 | 0.516 | 3.296 | 0.905 | 2.922 | 2.975 |

| **Fem # / Loc #** |  | **Variable** | **1** | **2** | **3** | **4** | **5** | **6** | **7** | **8** | **9** |
| --- | --- | --- | --- | --- | --- | --- | --- | --- | --- | --- | --- |
|  | **Taxon** |  | **BodyL** | **ScuL** | **ScuW** | **ETubH** | **ETubW** | **Chel1L** | **Chel1W** | **Chel2L** | **Chel2W** |
| 1 / 26 | *S. r. glorietus* |  | 2.13 | 1.742 | 1.736 | 0.224 | 0.089 | 0.333 | 0.206 | 0.576 | 0.222 |
| 2 / 26 | *S. r. glorietus* |  | 2.36 | 1.775 | 1.724 | 0.244 | 0.087 | 0.333 | 0.206 | 0.589 | 0.222 |
| 9 / 5 | *S. r. robustus* |  | 2.944 | 2.109 | 2.372 | 0.272 | 0.125 | 0.483 | 0.256 | 0.778 | 0.282 |
| 10 / 3 | *S. r. robustus* |  | 2.953 | 2.29 | 2.469 | 0.272 | 0.138 | 0.483 | 0.267 | 0.778 | 0.278 |
| 11 / 5 | *S. r. robustus* |  | 2.806 | 2.353 | 2.625 | 0.27 | 0.14 | 0.491 | 0.267 | 0.767 | 0.283 |
| 3 / 26 | *S. r. glorietus “trog”* |  | 2.175 | 1.82 | 1.95 | 0.2 | 0.102 | 0.37 | 0.213 | 0.7 | 0.224 |
| 4 / 26 | *S. r. glorietus “trog”* |  | 2.115 | 1.85 | 1.944 | 0.191 | 0.084 | 0.394 | 0.217 | 0.724 | 0.233 |
| 8 / 26 | *S. r. glorietus “trog”* |  | 1.95 | 1.63 | 1.72 | 0.2 | 0.09 | 0.472 | 0.2 | 0.667 | 0.224 |
| 5 / 1 | *Sclerobunus sp* |  | 2.325 | 2.145 | 2.305 | 0.231 | 0.12 | 0.441 | 0.233 | 0.761 | 0.256 |
| 6 / 4 | *S. r. robustus* |  | 3.125 | 2.33 | 2.3 | 0.249 | 0.136 | 0.533 | 0.264 | 0.817 | 0.289 |
| 7 / 4 | *S. r. robustus* |  | 2.719 | 2.25 | 2.463 | 0.267 | 0.143 | 0.557 | 0.272 | 0.847 | 0.302 |

| **Fem # / Loc #** |  | **Variable** | **10** | **11** | **12** | **13** | **14** | **15** | **16** | **17** | **18** |
| --- | --- | --- | --- | --- | --- | --- | --- | --- | --- | --- | --- |
|  | **Taxon** |  | **PalpFL** | **PalpFD** | **PalpPL** | **PalpPD** | **PalpTL** | **PalpTD** | **PalpTL** | **PalpTW** | **L1Troch** |
| 1 / 26 | *S. r. glorietus* |  | 0.634 | 0.279 | 0.38 | 0.217 | 0.468 | 0.233 | 0.428 | 0.156 | 0.272 |
| 2 / 26 | *S. r. glorietus* |  | 0.656 | 0.289 | 0.406 | 0.222 | 0.489 | 0.236 | 0.444 | 0.167 | 0.278 |
| 9 / 5 | *S. r. robustus* |  | 1.056 | 0.428 | 0.582 | 0.3 | 0.767 | 0.322 | 0.683 | 0.222 | 0.406 |
| 10 / 3 | *S. r. robustus* |  | 0.989 | 0.444 | 0.6 | 0.3 | 0.758 | 0.322 | 0.689 | 0.222 | 0.389 |
| 11 / 5 | *S. r. robustus* |  | 1.033 | 0.456 | 0.6 | 0.317 | 0.767 | 0.322 | 0.689 | 0.222 | 0.401 |
| 3 / 26 | *S. r. glorietus “trog”* |  | 1.159 | 0.316 | 0.617 | 0.249 | 0.833 | 0.244 | 0.75 | 0.176 | 0.35 |
| 4 / 26 | *S. r. glorietus “trog”* |  | 1.108 | 0.333 | 0.635 | 0.258 | 0.806 | 0.25 | 0.753 | 0.174 | 0.367 |
| 8 / 26 | *S. r. glorietus “trog”* |  | 1.006 | 0.289 | 0.578 | 0.233 | 0.75 | 0.233 | 0.689 | 0.172 | 0.344 |
| 5 / 1 | *Sclerobunus sp* |  | 0.944 | 0.396 | 0.558 | 0.268 | 0.711 | 0.289 | 0.672 | 0.2 | 0.356 |
| 6 / 4 | *S. r. robustus* |  | 1.19 | 0.448 | 0.672 | 0.322 | 0.9 | 0.389 | 0.794 | 0.222 | 0.406 |
| 7 / 4 | *S. r. robustus* |  | 1.175 | 0.492 | 0.683 | 0.336 | 0.889 | 0.361 | 0.811 | 0.244 | 0.4 |

| **Fem # / Loc #** |  | **Variable** | **19** | **20** | **21** | **22** | **23** | **24** | **25** | **26** | **27** |
| --- | --- | --- | --- | --- | --- | --- | --- | --- | --- | --- | --- |
|  | **Taxon** |  | **L1Fem** | **L1Pat** | **L1Tib** | **L1Met** | **L2Troch** | **L2Fem** | **L2Pat** | **L2Tib** | **L2Met** |
| 1 / 26 | *S. r. glorietus* |  | 0.747 | 0.389 | 0.569 | 0.63 | 0.3 | 1.06 | 0.441 | 0.9 | 0.943 |
| 2 / 26 | *S. r. glorietus* |  | 0.75 | 0.4 | 0.588 | 0.633 | 0.3 | 1.039 | 0.461 | 0.939 | 0.956 |
| 9 / 5 | *S. r. robustus* |  | 1.206 | 0.544 | 0.878 | 1.022 | 0.439 | 1.664 | 0.633 | 1.492 | 1.6 |
| 10 / 3 | *S. r. robustus* |  | 1.067 | 0.556 | 0.861 | 1.044 | 0.428 | 1.62 | 0.622 | 1.476 | 1.548 |
| 11 / 5 | *S. r. robustus* |  | 1.19 | 0.539 | 0.887 | 1.067 | 0.444 | 1.746 | 0.644 | 1.556 | 1.642 |
| 3 / 26 | *S. r. glorietus “trog”* |  | 1.313 | 0.533 | 1.009 | 1.067 | 0.41 | 2.06 | ? | ? | ? |
| 4 / 26 | *S. r. glorietus “trog”* |  | 1.341 | 0.547 | 1.006 | 1.08 | 0.411 | 2.093 | ? | ? | ? |
| 8 / 26 | *S. r. glorietus “trog”* |  | 1.206 | 0.522 | 0.922 | 0.986 | 0.378 | 1.926 | 0.567 | 1.55 | 1.571 |
| 5 / 1 | *Sclerobunus sp* |  | 1.109 | 0.533 | 0.806 | 0.978 | 0.356 | 1.68 | 0.583 | 1.476 | 1.608 |
| 6 / 4 | *S. r. robustus* |  | 1.294 | 0.617 | 0.978 | 1.213 | 0.433 | 1.88 | 0.683 | 1.744 | 1.9 |
| 7 / 4 | *S. r. robustus* |  | 1.311 | 0.594 | 1.006 | 1.222 | 0.434 | 1.94 | 0.698 | 1.74 | 1.896 |
