# Supplementary material for: Repeated and Time-Correlated Morphological Convergence in Cave-Dwelling Harvestmen (Opiliones, Laniatores) from Montane Western North America
Source: PLoS One. 2010 May 7;5(5):e10388. doi: 10.1371/journal.pone.0010388 (PMC2866537; doi:10.1371/journal.pone.0010388)
Supplement: Table S3 — PCR Primers. (0.04 MB DOC) [file pone.0010388.s004.doc]

| **Gene** | **Direction** | **PCR Primers** |
| --- | --- | --- |
| CO1 | Forward | C1-J-1510 5’-GGTCAACAAATCATAAAGATATTGG-3’ |
|  |  | C1-J-1517Spid 5’-AATCATARGGATATTGGAAC-3’ |
|  |  | C1-J-1718SpidA 5’-GGNGGATTTGGAAATTGRTTRGTTCC-3’ |
|  | Reverse | C1-2568 5’-GCTACAACATAATAAGTAtCATG-3’ |
|  |  | C1-2776Spider 5’-GGATAATCAGAATANCGNCGAGG-3’ |
| EF1-α | Forward | EF1-OP3 5’-TTTGARGAAATCCARAARGAAGT-3’ |
|  |  | EF1-OP4 5’-TACATYAAGAAGATTGGTTA-3’ |
|  |  | EF1-OP4SCLER 5’-TACATCAAGAAGATCGGTTA-3’ |
|  | Reverse | EF1-OPRC3PHAL 5’-ATGACCTGGGCRGTGAATTCTTC-3' |
|  |  | EF1-OPRC3SCLER 5’-ATGACCTGAGCCGTGAACTCTTC-3’ |
|  |  | EF1-OPRC4 5’-GAACTTGCANGCAATGTGAGC-3’ |
| 28S | Forward | ZX1 5’-ACCCGCTGAATTTAAGCATAT-3’ |
|  | Reverse | ZR2 5’-GCTATCCTGAGGGAAACTTCGG-3’ |
