# Supplementary material for: Repeated and Time-Correlated Morphological Convergence in Cave-Dwelling Harvestmen (Opiliones, Laniatores) from Montane Western North America
Source: PLoS One. 2010 May 7;5(5):e10388. doi: 10.1371/journal.pone.0010388 (PMC2866537; doi:10.1371/journal.pone.0010388)
Supplement: Table S4 — Model selection and testing results. (0.03 MB DOC) [file pone.0010388.s005.doc]

| **Analysis** | **Partitions** | **Model selected by AIC** | **-lnL Harmonic mean**  **(post burnin)** |
| --- | --- | --- | --- |
| CO1 | None (1) | GTR + I + G | -15502.40 |
|  | By codon position (3) | GTR + I + G (pos 1 and 2)  GTR + G (pos 3) | -14953.23 |
| 28S | None (1) | GTR + I + G | -6747.01 |
| EF1-α Exon | None (1) | GTR + I + G | -2075.54 |
|  | By codon position (3) | GTR + I (pos 1)  F81 (pos 2)  HKY + G (pos 3) | -2047.15 |
| CO1, 28S and EF1-α | By gene (3) | As above | -25397.69 |
|  | By gene and CO1, EF1-α codon (7) | As Above | -25191.16 |
