# Supplementary material for: Repeated and Time-Correlated Morphological Convergence in Cave-Dwelling Harvestmen (Opiliones, Laniatores) from Montane Western North America
Source: PLoS One. 2010 May 7;5(5):e10388. doi: 10.1371/journal.pone.0010388 (PMC2866537; doi:10.1371/journal.pone.0010388)
Supplement: Table S5 — Estimated divergence times from BEAST analyses (mean and confidence intervals). (0.04 MB DOC) [file pone.0010388.s006.doc]

| **Clade** | **3-CAL Tmrca Mean** | **3-CAL 95% min** | **3-CAL 95% max** | **2-CAL Tmrca**  **Mean** | **2-CAL 95%**  **min** | **2-CAL 95%**  **max** |
| --- | --- | --- | --- | --- | --- | --- |
| Root of tree | 60.91 | 43.65 | 78.66 | 66.22 | 47.32 | 87.65 |
| Travunioidea | 41.42 | 35.53 | 49.28 | 43.52 | 35.49 | 53.58 |
| *Sclerobunus* | 18.11 | 12.93 | 23.5 | 19.72 | 13.83 | 26.29 |
| *C. cavicolens* | 0.53 | 0.13 | 1.0 | 0.56 | 0.17 | 1.06 |
| *C. ungulatus* | 1.11 | 0.43 | 1.89 | 1.2 | 0.46 | 2.08 |
| Taos troglomorph | 5.43 | 2.86 | 8.17 | 5.94 | 3.04 | 9.04 |
| COTW/Mallory Cave | 4.91 | 2.64 | 7.32 | 7.09 | 3.7 | 10.71 |
| Fault Cave | 0.95 | 0.41 | 1.56 | 1.02 | 0.44 | 1.69 |
| Skeleton Cave | 0.69 | 0.28 | 1.19 | 0.75 | 0.28 | 1.25 |
