# Supplementary material for: Repeated and Time-Correlated Morphological Convergence in Cave-Dwelling Harvestmen (Opiliones, Laniatores) from Montane Western North America
Source: PLoS One. 2010 May 7;5(5):e10388. doi: 10.1371/journal.pone.0010388 (PMC2866537; doi:10.1371/journal.pone.0010388)
Supplement: Table S6 — Estimated rates of COI evolution as implied by 2-calibration and 3-calibration BEAST analyses. (0.04 MB DOC) [file pone.0010388.s007.doc]

**Table S6 - Estimated rates of COI evolution as implied by 2-calibration and 3-calibration BEAST analyses.**

|  | Observed COI divergence (uncorrected) | 2 calibration divergence time estimate (Ma) | 2 cal implied rate | 3 calibration divergence time estimate (Ma) | 3 cal implied rate |
| --- | --- | --- | --- | --- | --- |
| Taos *S. r. glorietus* (troglomorphic vs “typical” forms) | 8.7 | 5.9 | 1.47 | 5.4 | 1.61 |
| *S. nondimorphicus* vs. *S. r. idahoensis* | 7.63 | 4.0 | 1.91 | 3.9 | 1.96 |
| Fault Cave vs. Apex Cave *S. r. robustus* | 2.12 | 1.01 | 2.19 | 0.95 | 2.23 |

**Notes -** COI divergence values are summarized for nodes at three different time depths. From these values we calculated rates of COI evolution as implied by both 2- and 3-calibration BEAST trees. These rates vary across nodes (reflecting either saturation and/or among-lineage rate variation), but fall within a window of 1.5 – 2.25% pairwise divergence per million years, consistent with arthropod COI rates reported in the literature. Examples include Brower (1994) Rapid morphological radiation and convergence among races of the butterfly *Heliconius erato* inferred from patterns of mitochondrial DNA evolution. Proceedings National Academy of Sciences, USA 91: 6491-6495, and Pons et al. (2010) Nucleotide substitution rates for the full set of mitochondrial protein-coding genes in Coleoptera. Molecular Phylogenetics and Evolution *In Press*.
